# JMJD1C Exhibits Multiple Functions in Epigenetic Regulation during Spermatogenesis

**DOI:** 10.1371/journal.pone.0163466

**Published:** 2016-09-20

**Authors:** Ryusuke Nakajima, Hideyuki Okano, Toshiaki Noce

**Affiliations:** 1 Department of Physiology, Keio University School of Medicine, 35 Shinamomachi, Shinjuku-ku, Tokyo 160-8582, Japan; 2 Former: Mitsubishi-Kagaku Institute of Life Science, 11 Minami-Ooya, Machida, Tokyo, Japan; China University of Science and Technology, CHINA

## Abstract

*Jmjd1C* is one of the *Jmjd1* family genes that encode putative demethylases against histone H3K9 and non-histone proteins and has been proven to play an indispensable role in mouse spermatogenesis. Here, we analyzed a newly-bred transgenic mouse strain carrying a *Jmjd1C* loss-of-function allele in which a β-geo cassette was integrated into the intron of the *Jmjd1C* locus. *Jmjd1C* gene-trap homozygous testes exhibited malformations in postmeiotic processes and a deficiency in the long-term maintenance of undifferentiated spermatogonia. Some groups of spermatids in the homozygous testis showed abnormal organization and incomplete elongation from the first wave of spermatogenesis onwards. Moreover, histone H4K16 acetylation, which is required for the onset of chromatin remodeling, appeared to be remarkably decreased. These effects may not have been a result of the drastic decrease in gene expression related to the events but instead may have been due to the lack of interaction between JMJD1C and its partner proteins, such as MDC1 and HSP90. Additionally, significant decreases in *Oct4* expression and NANOG- and OCT4-expressing spermatogonia were found in the *Jmjd1C* homozygous mature testis, suggesting that JMJD1C may participate in the maintenance of spermatogonial stem cell self-renewal by up-regulating *Oct4* expression. These results indicate that JMJD1C has multiple functions during spermatogenesis through interactions with different partners during the spermatogenic stages.

## Introduction

Germ cells in mammals are the only cell lineage to transmit species-specific genetic and epigenetic information from one generation to the next. Therefore, the lineage leading to gamete production provides a simple but excellent model system to understand the molecular basis underlying every developmental event. Spermatogenesis features three major processes (maintenance of self-renewing stem cells, cyclic and well-organized differentiation through meiosis and maturation accompanying global chromatin remodeling). These processes are held under strict control via the systematic regulation of gene expression, epigenetic modification and genome DNA integrity. Among these processes, the methyltransferases and demethylases responsible for maintaining the dynamic balance associated with the methylation of lysine (K) residues of core histones have been demonstrated to play a central role in the epigenetic modification of transcription and chromatin structures during spermatogenesis [1, 2]. For instance, *G9a*, which encodes the H3K9 methyltransferase, and *Jmjd1A*, which encodes the H3K9 demethylase, play essential roles in the regulation of the balanced control of spermatogenesis-specific gene expression by methylated H3K9 [3, 4]. However, recent studies have revealed that many histone methylation and demethylation enzymes are not only specific for the K residues of core histones but also act on the K residues of non-histone proteins. Indeed, a methylated K residue is found in more than 400 non-histone proteins located both inside and outside of the nucleus; these proteins are involved in diverse biological events and diseases [5]. Moreover, the finding that *G9a* plays roles in the methylation of a histone acetyltransferase (HDAC1) and DNA methyltransferase (DMNT1) indicates that methyl modification consists of a comprehensive network that cooperates with other epigenetic regulation mechanisms [5].

Based on our microarray analysis that compared the expression levels of *JmjC* domain family genes between ES cells, the inner cell mass of blastocysts, epiblasts, and primordial germ cells (PGCs) from E8.5 embryos, we focused on the *Jmjd1* family *Jmjd1C* gene (also known as Kdm3C and Trip8) because its expression level was much higher in ES cells and PGCs than cells of other stages (data not shown). Then, the *Jmjd1C* ortholog *Jmjd1A* (*Kdm3A*), which encodes a demethylase to remove mono/di-methyl modifications from histone H3K9, was revealed for the first time to play an essential role in spermatogenesis. A detailed analysis of the spermatogenic deficiency in *Jmjd1A* gene-trap mice demonstrated that JMJD1A participated in the transcriptional control of *Transition protein 1* (*Tnp1)* and *Protamine 1* (*Prm1)* through H3K9 demethylation of the upstream promoter regions, leading to postmeiotic chromatin condensation [6]. However, JMJD1A is known to have other functions in metabolic syndrome and sex determination [7, 8]. During the progress of our work, a precedent study on *Jmjd1C*-deficient mice was reported that showed the male infertility phenotype of *Jmjd1C* null-knockout mice due to the progressive reduction of germ cells after 3 months of age, indicating that JMJD1C contributed to the long-term maintenance of male germ cells [9]. A similar phenotype involving the progressive loss of proliferating spermatogonia was reported in *Promyelocytic leukaemia zinc finger* (*Plzf*, also known as *Zfp145* and *Zbtb16*)-knockout mice; the expression of this gene is restricted to undifferentiated spermatogonia [10]. The germ cell degeneration due to the *Plzf* defect starts at 2 weeks of age, which is apparently much earlier than the *Jmjd1C* defect; thus, it is unlikely that the progress of germ cells found in the mature testis of *Jmjd1C*-deficient mouse is due only to the loss of *Plzf* expression. Furthermore, recent studies reported the contradictory finding that JMJD1C had no enzymatic histone H3K9 demethylation activity [9, 11], which raised a possibility that *Jmjd1C* might play a non-enzymatic role, such as a serving as a scaffold for the construction of a regulatory apparatus, rather than contributing its enzymatic activity alone. In this study, we investigated the spermatogenic deficiency observed in *Jmjd1C* gene-trap mutant mice and revealed that *Jmjd1C* had multiple functions in chromatin remodeling during spermiogenesis and the maintenance of spermatogonial stem cells that were accompanied by multiple partner proteins.

## Materials and Methods

### Generation of *Jmjd1C*^*gt/gt*^ mice and genotyping

Germline transmission (heterozygous) mice generated from a *Jmjd1C* gene-trap ES cell clone (TG Resource Bank, clone #8131) were purchased from TransGenic Inc. (Fukuoka, Japan). The heterozygous mice (*Jmjd1*^*+/gt*^) were backcrossed with 129/svj and Jcl:ICR mice. Homozygous (*Jmjd1C*^*gt/gt*^) mice were then generated by intercrossing *Jmjd1C*^*+/gt*^ mice and identified by PCR based genotyping using the allele-specific primer pairs listed in S1 Table. 10, 94, 24, 8 and 38 mice were used for flow-cytometry, histochemical, immunoblotting, immunoprecipitation, experiments and RNA/DNA analyses, respectively, and total approximately 300 mice including ones for the maintenance of the strain were used for this study. Animal experiments was fully approved by the Keio University Institutional Animal Care and Use Committee (approval number: 12023–2) and all animal care and experimental procedures were performed in accordance with the Institutional Guidelines on Animal Experimentation at Keio University.

### Testicular cell preparation and separation by flow cytometry

After treatment with collagenase type IV (Sigma), the testicular tubules were dissociated with a 0.05% Trypsin-EDTA solution (Sigma). The resulting single cell suspensions were used for cell fractionation, nuclear isolation and cell sorting. Subcellular fractionation was performed with the Subcellular Protein Fractionation Kit for Tissues (Thermo Scientific) according to the manufacturer’s instruction. For flow-cytometry, the cells were stained with Hoechst 33342 and then analyzed with the FACS Aria III (BD Biosciences).

### RNA preparation and QRT-PCR

Total RNA was prepared from mouse tissues with the Qiagen RNeasy kit or the Sepasol-RNAI SuperG kit (Nacalai Tesque, Inc.) and reverse transcribed using an oligo-dT primer and a Superscript III first-strand cDNA synthesis kit (Invitrogen) in accordance with the manufacturers’ instructions. The real time (RT)-PCR was performed with Ex-Taq (Takara), and the quantitative real-time (QRT) PCR was analyzed using the SYBR Premix EX TaqII kit (Takara) and ViiA7 (Applied Biosystems). The QRT-PCR reactions were normalized against the β-actin transcript levels. The primer pairs used for the cDNA analysis are listed in S1 Table.

### Immunohistochemical staining, X-gal staining and immunoblotting analysis

For immunohistochemistry, the testes and other tissues were fixed with either Bouin’s fixative or 4% Paraformaldehyde (PFA) phosphate buffer solution and embedded in paraffin. After de-paraffinization, 5 μm-thick sections were stained with Hematoxylin or treated with Target Retrieval Solution (Dako, S1699) for unmasking, blocked with 3% bovine serum albumin (BSA) /0.1% Triton X-100, and then reacted with primary and secondary antibodies as previously described [12]. Fluorescent immunohistochemical images were obtained with an Olympus IX71 and the MetaMorph software. For X-gal staining, the tissues were fixed with 0.2% glutalaldehyde/2% formalin buffer and then colorized according to the X-gal staining protocol. For the immunoblotting analysis, testicular cell extracts (15 μg per lane) were separated by SDS-PAGE. The blotted membranes (Immobilon-XP) were immunoreacted with primary antibodies and visualized with the ECL Prime Western blotting Detection kit (GE Healthcare) containing secondary antibodies, HRP (horseradish peroxidase)-labeled anti-mouse IgG and anti-rabbit IgG, and the Quant LAS4000-mini (GE Healthcare) in accordance with the manufacturer’s instruction. The antibodies used for immunofluorescence and immunoblotting detection are listed in S2 Table.

### Immunoprecipitation

Immunoprecipitation (IP) with an antibody against MDC1 was performed using the Protein G HP SpinTrap (GE Healthcare) according to the manufacturer’s instruction. Testicular cell lysates were prepared with RIPA lysis buffer (10^7^ cells/ml). Then, 200 μl of each lysate was reacted with Protein G Sepharose beads pretreated with 2 μg of anti-MDC1 or normal rabbit IgG. IP proteins were extracted from the precipitated beads by the addition of 50 μl of 2x SDS sample buffer. A total of 8 μl of each IP sample and 2 μl of lysate for input per lane were applied for the SDS-PAGE.

## Results

### Spermatogenic deficiency observed in *Jmjd1C* gene-trap mice

**Fig 1:**
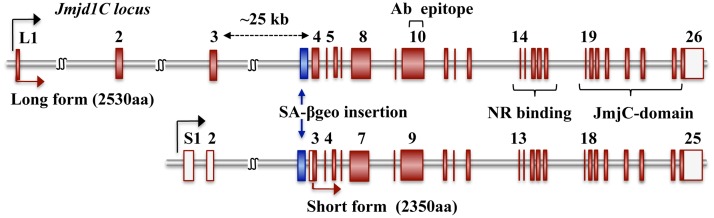
Two transcript variants in the *Jmjd1C* gene-trap allele. Schematic representation of the *Jmjd1C* exon/intron constructs of the long-form (XM006513035.1) and short-form (XM001242396.1) variants. The blue box shows a SA-β-geo cassette integrated within the intron. The antibody (Ab) epitope site used in this study, the nuclear receptor (NR) binding region and Jmj-C domain coding region, are indicated.

**Fig 2:**
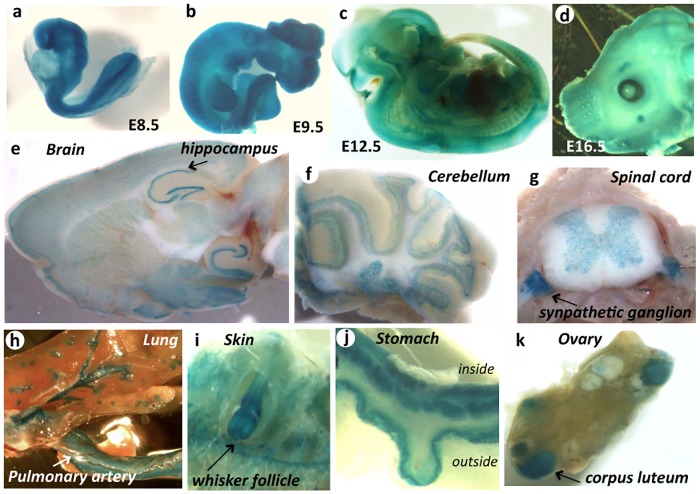
Expression profile of *Jmjd1C* detected with X-Gal staining of *Jmjd1C*^*+/gt*^ mice. X-gal staining images of whole embryos at E9.5 (a), E10.5 (b), and E12.5 (c) and a facial portion of the E16.5 embryo (d) are shown. In adult tissues, cross sections of the brain (e), cerebellum (f), spinal cord with sympathetic ganglions (g), lung with pulmonary artery (h), whisker follicle (i), walls of stomach (j) and ovary (k) are stained with X-gal solution.

**Fig 3:**
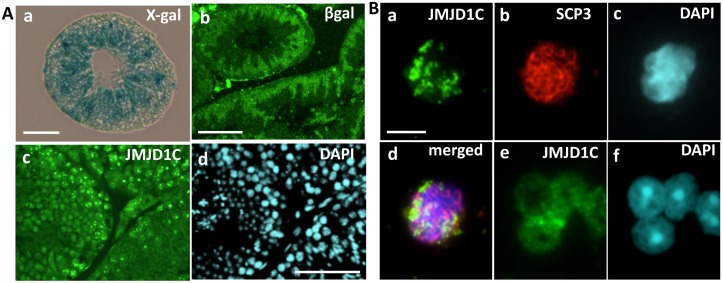
JMJD1C expression in mouse testicular germ cells. A) X-gal staining (a) and immunostaining with anti-β-galactosidase (b) in cross-sections of adult *Jmjd1C*^*+/gt*^ mouse testes (4-month-old). The adult testis (4-month-old) sections (tubules at the stage VII and X) were immunostained with anti-JMJD1C (c) and DAPI (d). Scale bars, 100 μm. B) Spermatocyte nuclei were prepared from cells dissociated from adult testis and stained with anti-JMJD1C (a), anti-SCP3 (b), DAPI (c) and a merged image (d). Spermatids were also stained with anti-JMJD1C (e) and DAPI (f). Scale bar in (a) for (a)-(f), 10 μm.

Two major *Jmjd1C* transcript variants have been demonstrated to be driven by alternative promoters. The long-form transcript encodes a 2530 amino acid protein that is 180 amino acids longer than the short-form protein (2350 amino acids) at the N-terminal end. The *Jmjd1C*-deficient mouse line (*Jmjd1C* ^*gt/gt*^) used in this study was generated from a gene-trap ES cell clone carrying a mutation allele in which a SA(splicing acceptor)-β-geo cassette was inserted into the third intron of the long-form and the second intron of the short-form protein; the cassette was integrated approximately 1.0 kb upstream of the next exon (Fig 1). Theoretically, the β-geo insertion interrupts *Jmjd1C* transcription of both variants and the β-galactosidase translation products precisely reflect the *Jmjd1C* transcription activity in vivo, which provides us with a convenient and powerful tool to evaluate the *Jmjd1C* expression patterns in embryos and whole adult tissues. X-gal staining of *Jmjd1C* ^*+/gt*^ mice showed cell types with various *Jmjd1C* expression patterns. In mid-gestation embryos (E8.5 onward) undergoing organogenesis, major expression was detected in the neural fold (Fig 2a and 2b). The strong expression in neural cells continued throughout embryogenesis and extended to the brain, spinal cord and sympathetic ganglions during adulthood. Additionally, notable expression was observed in tissues such as the heart, blood vessels, whisker follicles, and stomach (Fig 2c–2j). *Jmjd1C* expression in the testis, where the X-gal staining intensity appeared to be much lower than neurons, was primarily detected in the inner cell layers of the seminiferous tubule in which meiotic and postmeiotic cells were located (Fig 3Aa). Meiotic and postmeiotic cell expression was confirmed by immunostaining with anti-β-gal and anti-JMJD1C antibodies (Fig 3Ab and 3Ac). Additionally, we found that JMJD1C localized to a part of the chromatin in the nuclei of Synaptonemal Complex Protein (SCP3)-positive spermatocytes (Fig 3B). Conversely, X-gal staining of the adult ovary showed that *Jmjd1C* expression was primarily detected in the corpus luteum and was barely detected in the oocytes (Fig 2k).

**Fig 4:**
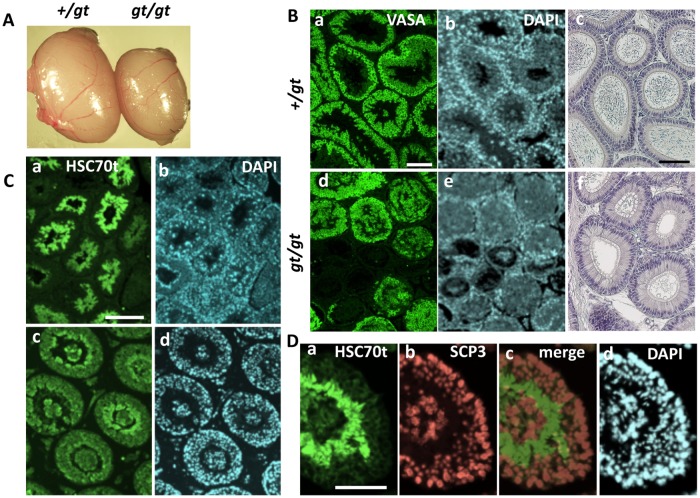
Comparison of homozygote (gt/gt) testes with heterozygote (+/gt) testes. A) Heterozygote (+/gt) and homozygote (gt/gt) testes removed from 4-month-old littermate mice were compared. B) Histological sections of +/gt (*Jmjd1C* ^*+/gt*^) heterozygote (a, b) and gt/gt (*Jmjd1C* ^*gt/gt*^) homozygote (d, e) testes from 4-month-old littermate mice were stained with anti-VASA (MVH) (a, d), which recognized testicular germ cells at all spermatogenic stages, and DAPI for nuclear staining (b, e). Epididymis sections of the same +/gt (c) and gt/gt (f) littermate mice were counter-stained with Hematoxylin. Scale bars in (a) for (a, b, d, f) and in (c) for (c, f) are 100 μm. C) Testis sections from +/gt (a, b) and gt/gt (c, d) 2-month-old littermate mice were immunostained with anti-HSC70t, which specifically reacted with round and elongated spermatids. Section of a +/+ littermate testis stained with anti-HSC70t showed almost the same staining image with that of +/gt testis (data not shown). DAPI staining is shown in the same fields (b, d). Scale bar in (a) for (a-d), 100 μm. D) Section of a gt/gt testis double-stained with anti-HSC70t (a) and anti-SCP3 (b), which specifically recognized spermatids and spermatocytes, respectively. (c) is a merged image of (a+b), shown together with nuclear-staining with DAPI (d). Scale bar in (a) for (a-d), 100 μm.

Despite the wide range of *Jmjd1C* expression from early embryos to adult tissues, the homozygous mice (*Jmjd1C*^*gt/gt*^) were normal in appearance and the abnormality was only found in the testes. Consistent with the previous report of null-knockout mice [9], the testes in *Jmjd1C*^*gt/gt*^ mice 2–3 months of age appeared to be slightly smaller than the testes from the heterozygote (*JmjdC*^*+/gt*^) and wild-type mice (Fig 4A). Cross-sections of the adult testis and epididymis showed a remarkable loss of testicular germ cells expressing the VASA (also known as MVH and DDX4) protein [12] and few spermatozoa in the *Jmjd1C*^*gt/gt*^ mice (Fig 4B). The previous report noted that the *Jmjd1C* deficiency appeared only during the long-term maintenance of spermatogonial stem cells [9]. However, in addition to the progressive loss of germ cells, we found another deficiency during spermatogenesis of the *JmjdC*^*gt/gt*^ testis. Cross-sections of the *Jmjd1C* ^*gt/gt*^ adult testis (4-month-old) stained with spermatid-specific anti-Heat Shock Cognate protein 70t (HSC70t) [13] clearly showed the existence of disorganized stratification with abnormally shaped spermatids at the edge of the lumen (Fig 4C). Additionally, double-staining with anti-HSC70t and spermatocyte-specific anti-SCP3 showed the presence of cell aggregates consisting of spermatocytes and spermatids, which were completely detached from the spermatogenic cell layers in many parts of the seminiferous tubules (Fig 4D). These findings strongly suggest the importance of *Jmjd1C* expression for the completion of spermatogenesis in the meiotic and postmeiotic stages.

**Fig 5:**
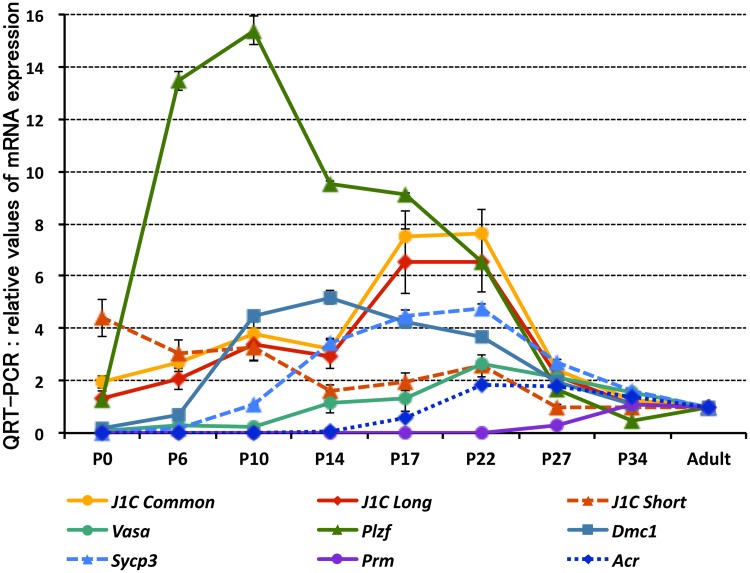
Expression kinetics of *Jmjd1C* variants and selected spermatogenic marker genes in testicular development. Single-stranded cDNA was prepared from the testes at the indicated days after birth. The expression levels of the *Jmjd1C* variants and selected genes were quantitatively analyzed with *Jmjd1C* variant- and gene-specific primer pairs. The values indicated were the relative values of each gene expression level in the adult (4-month-old) testis (set as 1.0) after standardization with the β-actin expression level in each testis. Error bars indicate the SEM (n = 4).

Examination of the *Jmjd1C* expression kinetics during the first wave of spermatogenesis showed that *Jmjd1C* short form expression was highest at birth and then gradually decreased with development, whereas expression of the long form reached its peak around the onset of meiosis, corresponding with the expression of *Scp3* and *Vasa* (Fig 5). Most of the *Jmjd1C* transcripts appeared to be the long-form variant during testis development (S1A Fig), indicating that the majority of *Jmjd1C* transcription in germ cells from the meiotic stage onwards was driven by the long-form promoter. The high level of *Jmjd1C* expression in the meiotic and postmeiotic cells was also confirmed at the protein level (S1B Fig). Here, although the anti-JMJD1C antibody used in this study was raised against an oligo-peptide located within exon 10 that was common between both the short and long forms (Fig 1), we barely detected a short-form protein band by immunoblotting.

**Fig 6:**
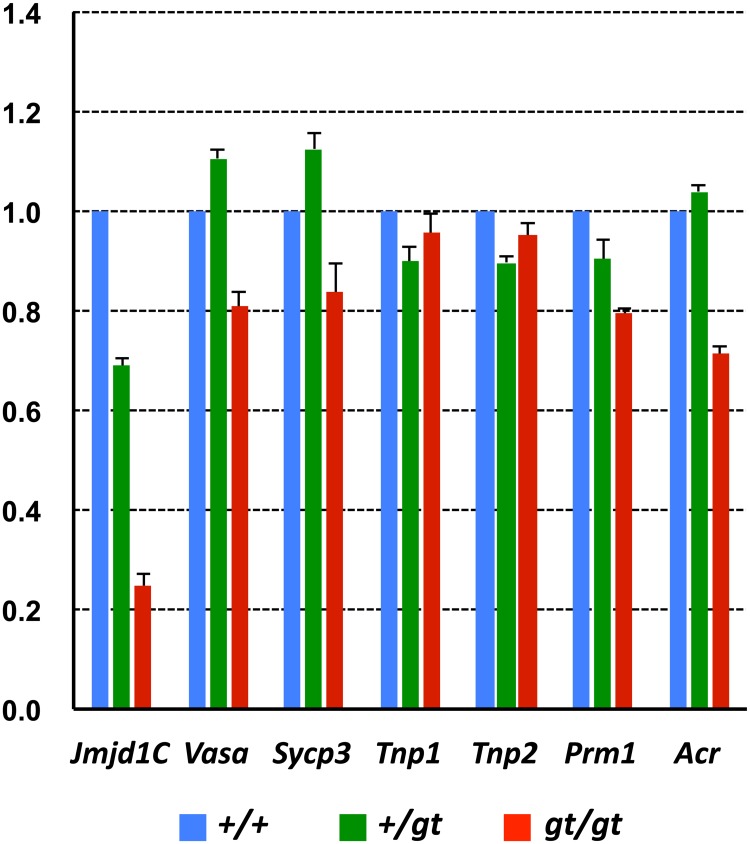
QRT-PCR analyses showing differences in gene expressions between the +/+, +/gt, and gt/gt testes. Single-stranded cDNA was prepared from the +/+, +/gt and gt/gt testes of adult (2-month-old) littermate mice. Expression levels of total *Jmjd1C* (short + long variants) and selected spermatogenesis marker genes were compared. The values with SEM (n = 4) were the relative gene expression values in the +/+ testis (set as 1.0) after standardization with the β-actin expression level in each testis.

When the difference in *Jmjd1C* expression was examined between genotypes, we found that our *Jmjd1C*^*gt/gt*^ mouse was not completely null for *Jmjd1C* expression. The quantitative real time (QRT)-PCR analysis indicated that the *Jmjd1C*^*+/gt*^ and *Jmjd1C*^*gt/gt*^ testes expressed *Jmjd1C* transcripts at levels that were nearly 60% and 20% of the levels in the wild-type testis, respectively (Fig 6 and S2A Fig). Indeed, a small amount of full-length JMJD1C protein was detectable in the *Jmjd1C* ^*gt/gt*^ testis by immunoblotting (S2B Fig). Therefore, we think it is feasible that a small amount of wild-type transcript is produced when a *β-geo* cassette is integrated within the intron region of a target gene, possibly by alternative splicing of the read-through precursor transcript containing the inserted β-geo cassette. A similar phenomenon was reported in *Jmjd1A*-deficient mice [6] in which even an incomplete knockout led to the same phenotype as the null-type knockout. Therefore, *Jmjd1C* is likely to have a quantitative threshold for completing its function that is probably at least 20% greater than the wild-type level. In contrast, the QRT-PCR analysis did not reveal drastic changes in the expression levels of selected spermatogenesis marker genes, such as *Scp3*, *Vasa*, and *Prm1* (Fig 6). These results suggest that JMJD1C is unlikely to have a direct effect on the transcriptional regulation of these germ cell-specific genes.

**Fig 7:**
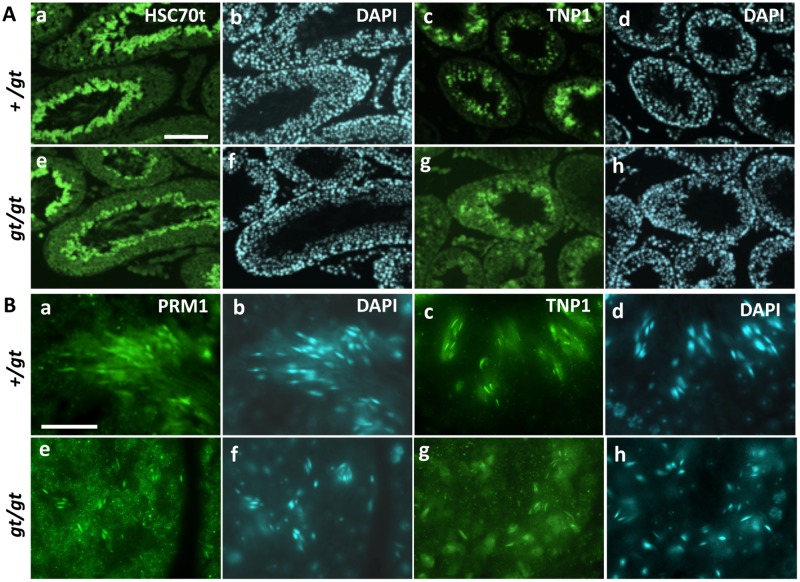
Spermatogenic abnormalities found in young mature testis from *Jmjd1C* ^*gt/gt*^ mice. A) Sections of littermate +/gt (a-d) and gt/gt (e-h) testes on postnatal day 54 (stage XII) were stained with antibodies against the spermatid marker proteins anti-HSC70t (a, e) and anti-TNP1(c, g). DAPI stained images corresponding to each immunostaining are shown in (b, d, f, h). Scale bar in (a) for (a-h), 50 μm. Section of a +/+ littermate testis showed almost the same staining image with those of +/gt testis (data not shown). B) Sections of littermate +/gt (a-d) and gt/gt (e-h) testes on postnatal day 30 stained with anti-PRM1 (a, e) and anti-TNP1(c, g) are shown together with DAPI staining. Scale bar in (a) for (a-h), 20 μm.

Based on their appearance, the *Jmjd1C* ^*gt/gt*^ testes from mice at two months of age were not different from the testes of their littermate controls. Nevertheless, cross-sections stained with antibodies against spermatid-specific proteins revealed a subtle contrast between the *Jmjd1C* ^*gt/gt*^ and control mice (Fig 7). When sections of the wild-type and *Jmjd1C* ^*+/gt*^ testes from postnatal day 54 mice were stained with anti-HSC70t and ant-TNP1, the positively stained spermatids lining the lumen were localized in a well-ordered array. In contrast, in the *Jmjd1C* ^*gt/gt*^ testis of the littermate, the positively stained spermatids were located in a disarrayed manner and the elongation of the spermatids appeared incomplete (Fig 7A). Similar abnormalities in the spermatid arrangement were observed in the *Jmjd1C* ^*gt/gt*^ testis at postnatal day 30 at the final stage of first wave spermatogenesis. Newly generated sperm heads were radially arranged in the *Jmjd1C* ^*+/gt*^ testis but randomly localized in the *Jmjd1C* ^*gt/gt*^ testis (Fig 7B and S3 Fig). Thus, the morphogenetic disorganization found in *Jmjd1C*-deficient spermatogenesis may be the cause of the clustered detachment of spermatogenic cells at the later stage, as shown in Fig 4D.

### Chromatin remodeling abnormality during spermiogenesis

**Fig 8:**
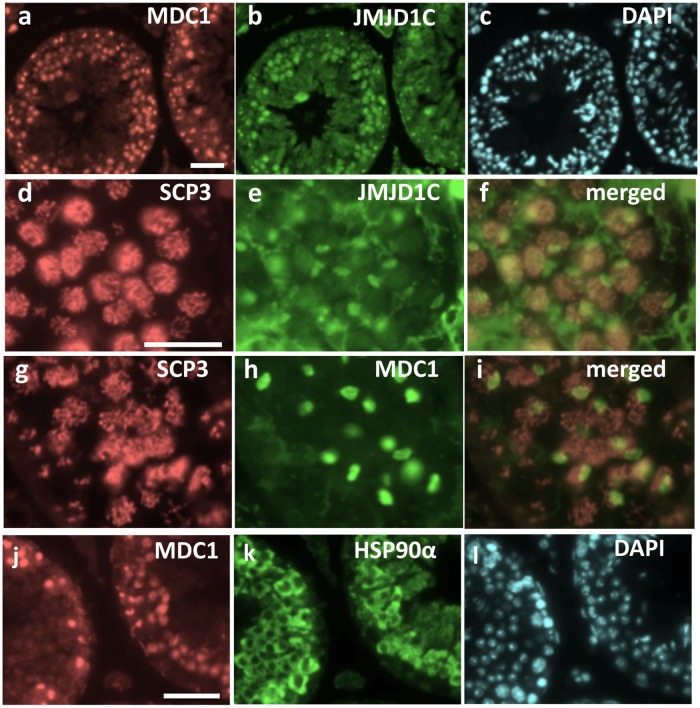
Co-localization of the JMJD1C, MDC1, RNF8 and HSP90 proteins in spermatogenic cells. Each section of a wild-type adult testis (4-month-old) was double-stained with antibodies against MDC1 and JMJD1C (a-c), SCP3 and JMJD1C (d-f), SCP3 and MDC1 (g-i), and MDC1 and HSP90α (j-l). Particles strongly stained with anti-JMJD1C (e) and anti-MDC1 (h) found in SCP3-positive spermatocyte nuclei are XY bodies. (f) and (i) are merged images of (d+e) and (g+h), respectively. (c) and (l) are DAPI stained images of (a, b) and (j, k), respectively. Scale bars in (a) for (a-c), in (d) for (d-i), and in (j) for (j-l) are 50 μm, 25 μm and 50 μm, respectively.

Regarding the enzymatic activity of JMJD1C itself, several reports have provided negative evidence against demethylase activity for histone H3K9 [9, 11]. Instead, demethylase activity for non-histone protein(s) has been recently reported. MDC1 (Mediator of DNA damage check-point protein 1), which is a core factor of the DNA damage response (DDR) complex, was first identified as a non-histone target protein of demethylation by JMJD1C [14]. The well-defined study demonstrated that JMJD1C-dependent demethylation at MDC1 K45 was not required for its function in the DDR. Mice lacking *Mdc1* were demonstrated to undergo meiotic arrest at the mid-pachytene stage, leading to male sterility. Additionally, MDC1 plays an indispensable role in the formation and function of the XY body, which is a meiosis-specific structure formed around the paired X/Y chromosomes in a spermatocyte [15]. Staining of sections with antibodies against MDC1 and JMJD1C showed the co-localization of both proteins in the XY body (Fig 8), corresponding to their functional cooperation during the meiotic processes. However, the *Jmjd1C* ^*gt/gt*^ testis did not show the meiotic arrest found during MDC1 deficiency and did not exhibit abnormalities in MDC1 localization and XY body formation (S4A Fig), suggesting that JMJD1C was not essential for MDC1-mediated XY body formation. JMJD1C has not been demonstrated to promote an interaction between MDC1 and another core factor, Ring finger protein 8 (RNF8); these three factors bind to one another to form a core DDR complex [14]. Interestingly, mice lacking *Rnf8* (encoding an ubiquitin ligase) also exhibited spermatogenic deficiency, which caused an imperfection at the postmeiotic stage similar to the *Jmjd1C*^*gt/gt*^ phenotype described above [16]. The Rnf8 defect was revealed to appear during the histone-protamine transition process via histone H4K16 acetylation [16]. However, RNF8 primarily localizes in spermatocytes and showed no remarkable changes in the *Jmjd1C* ^*gt/gt*^ testis (S4B Fig).

**Fig 9:**
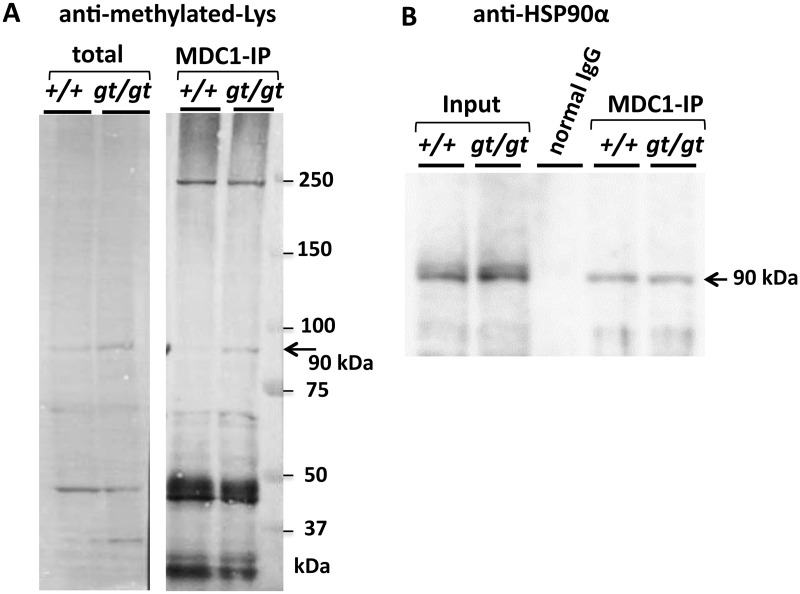
Detection of methylated non-histone proteins is affected by *Jmjd1C* deficiency. A) Total extracts of 15 μg were prepared from +/+ and gt/gt testes of littermate mice. IP samples obtained using combinations of anti-MDC1 and +/+ or gt/gt testis lysates were applied for SDS-PAGE (10%), blotted and then detected with anti-methylated-Lys in conjugation with HRP-labeled anti-rabbit IgG. An arrow indicates a 90 kDa protein band. B) MDC1-IP samples from +/+ and gt/gt testes (A), control IgG-IP and the input lysates from the +/+ and gt/gt testes were immunoblotted. The membrane was reacted with anti-HSP90α and then visualized with HRP-anti-rabbit IgG and the detection kit.

Although the immunostaining of testis sections with anti-methylated lysine showed no differences between the *Jmjd1C* genotypes (S4C Fig), immunoblotting analysis with the same antibody revealed several non-histone protein bands with significantly increased methylation levels in the *Jmjd1C* ^*gt/gt*^ testis. A band representing an approximately 90 kDa protein appeared to be hypermethylated in the total extract from the *Jmjd1C* ^*gt/gt*^ testis; this band was also detected as a hypermethylated protein immunoprecipitated (IP) with anti-MDC1 from the *Jmjd1C* ^*gt/gt*^ testis (Fig 9A). Judging from the size and the lysine-methylated modification, the hypermethylated protein should be HSP90. Indeed, we found that HSP90 was immunoprecipitated with anti-MDC1 (Fig 9B) and co-localized with MDC1 in spermatogenic cells (Fig 8j–8l and S4A Fig).

**Fig 10:**
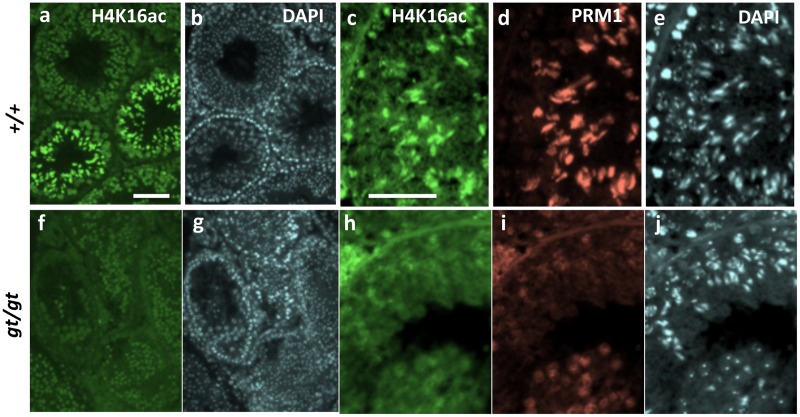
Abnormal histone-protamine transition found in the *Jmjd1C* ^*gt/gt*^ testis. Testis sections of littermate 4-month-old +/+ (a-e) (stage XI) and gt/gt (f-j) testis were immunostained with anti-acetylated histone H4K16 (a, f) or double-stained with anti-acetylated histone H4K16 (c, h) and anti-PRM1 (d, i). (b, g, e, j) represent DAPI staining of the corresponding fields. Scale bars in (a) for (a, b and f, g) and in (c) for (c-e and h-j) are 100 μm and 50 μm, respectively.

**Fig 11:**
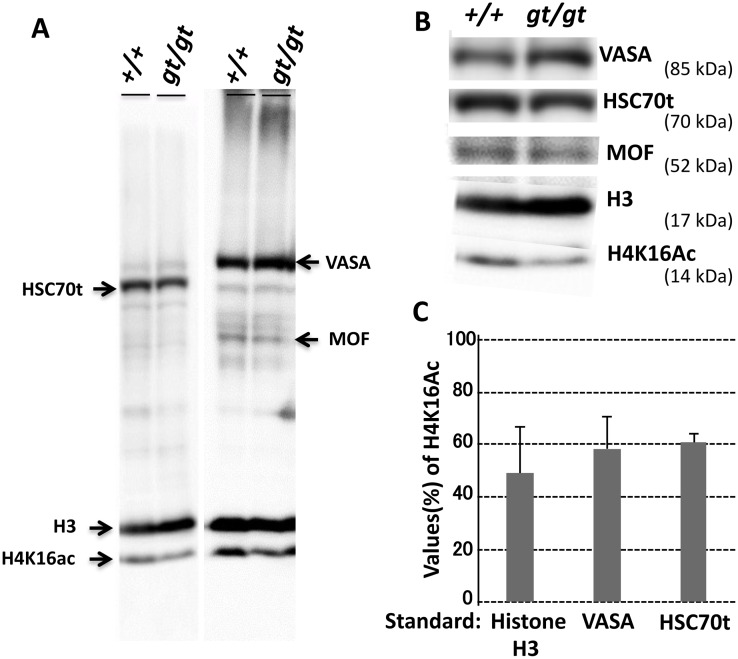
Detection of hypo-acetylated K16 on histone H4 in the *Jmjd1C* ^*gt/gt*^ testis. Approximately 15 μg protein extracts from +/+ and gt/gt testes (postnatal day 56 littermates) were applied on SDS-PAGE (10–20% gradient gel) and blotted onto membranes. A) The membranes were reacted with a mixture of three antibodies against HSC70t, histone H3 (H3) and acetylated H4K16 (H4K16Ac) (left) or four antibodies against VASA, MOF, histone H3 and acetylated H4K16 (right) and then detected with HRP-conjugated anti-IgG and an ECL detection reagent. Specific reactant of each antibody used in this mixed immunoblotting is shown in S5 Fig. B) Comparison of the detected protein bands between the +/+ and gt/gt extracts. C) Semi-quantitative comparison of the acetylated H4K16 bands between the +/+ and gt/gt testes extracts. Values obtained from the +/+ and gt/gt testes were normalized with those of the histone H3, VASA and HSC70t protein bands, which roughly corresponded to the numbers of total cells, germ cells at stages from spermatogonium to late spermatid, and spermatids, respectively. Bars in the graph show relative ratios (%) of values of the H4K16ac band in gt/gt against those in +/+ after normalizing with values of standard protein bands as indicated below. Error bars indicate the SEM (n = 4–6).

**Fig 12:**
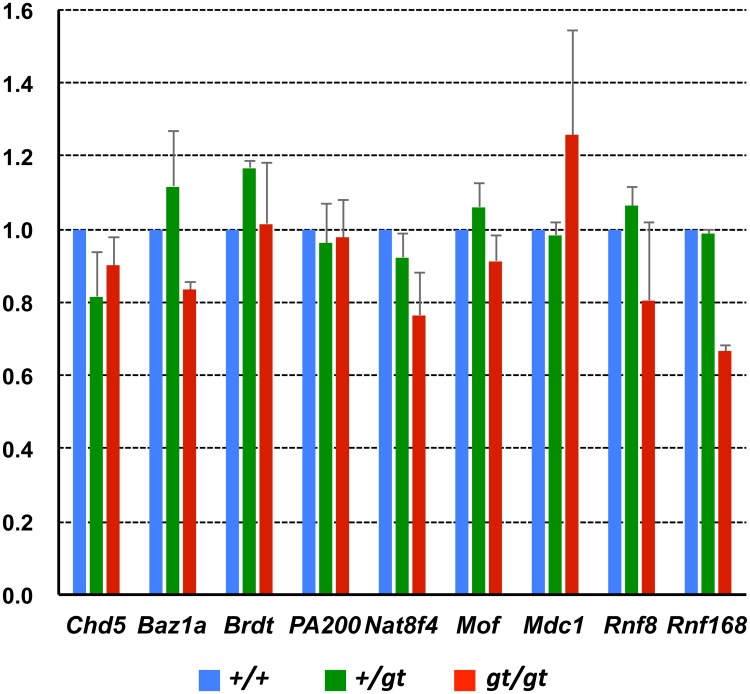
QRT-PCR analyses of selected genes related to chromatin remodeling between the +/+, +/gt, and gt/gt testes. Single-stranded cDNA was prepared from the +/+, +/gt and gt/gt testes of adult (2-month-old) littermate mice. Expression levels of selected genes related to chromatin remodeling were compared. The values with SEM (n = 4) were the relative gene expression values in the +/+ testis (set as 1.0) after standardization with the β-actin expression level in each testis.

Based on this line of analysis, we examined histone H4K16 acetylation (H4K16ac) in the *Jmjd1C*^*gt/gt*^ testis. Immunostaining with anti-H4K16ac showed a remarkable decrease in the *Jmjd1C*^*gt/gt*^ adult testis compared with the littermate controls (Fig 10). The significant decrease in H4K16ac in the *Jmjd1C* ^*gt/gt*^ testis was validated by the quantitative analysis of the protein bands detected with the anti-H4K16ac antibody, with the values of the histone H3, VASA or HSC70t bands corresponding to the number of total cells, germ cells or spermatids, respectively, that were utilized as the normalization standards (Fig 11 and S5 Fig). Furthermore, although immunostaining with anti-PRM1 clearly revealed expression in the spermatid nuclei of the control littermates, the staining signals were barely observed in the spermatid nuclei from the *Jmjd1C* ^*gt/gt*^ testis (Fig 10). Regarding histone H4K16 acetylation during spermiogenesis, *Mof* (*Kat8*), which encodes an acetyltransferase required for DDR, and *Nat8f4* (1700019G17Rik), which encodes a testis-specific putative acetyltransferase, are responsible for H4 hyper-acetylation in a ubiquitination-dependent and -independent manner, respectively [17, 18]. However, MOF expression showed no apparent difference in localization between the testes of *Jmjd1C* wild-type and homozygous littermate mice (S4D Fig). Additionally, the expression levels of selected genes related to the core histone removal (*Rnf8*, *Rnf168*, *Mdc1*, *Nat8f4* and *Mof*), chromatin remodeling (*Chd5*, *Brdt* and *Baz1a*) and proteasome activation (*PA200*, also known as *Psme4*) pathways in spermatids showed no remarkable differences between the testes of the three different *Jmjd1C* genotypes, whereas a somewhat moderate reduction was detected in *Rnf8* and *Rnf168* (Fig 12) [15, 16, 19–24]. Altogether, these results suggest that *Jmjd1C* has an indirect function in the regulation of histone H4K16 hyper-acetylation that leads to histone-protamine remodeling during sperm formation, although the action mechanism remains undefined.

### Deficiency in the maintenance of spermatogonial stem cells (SSCs)

Previous reports demonstrated that *Jmjd1C* deficiency caused an abnormality in the long-term maintenance of spermatogonial stem cells (SSCs) leading to the degeneration of testicular tubules after 3 months of age [9]. Almost the same testis degradation time course was observed in the testis from our *Jmjd1C* ^*gt/gt*^ mouse (Fig 4). The developmental kinetics of *Jmjd1C* expression showed that the short-form variant of *Jmjd1C* was predominantly expressed in spermatogonia containing SSCs (Fig 5). The JMJD1C function related to stem cell potential has been well investigated in pluripotent ES cells. For instance, OCT4 (POU5F1) and primarily the short form of JMJD1C bind to one another’s promoter regions to activate transcription in a positive feedback-like loop, and *Nanog* expression promoted by an OCT4 and JMJD1C compound plays a critical role in the maintenance of pluripotent stem cell-ness [17, 25].

**Fig 13:**
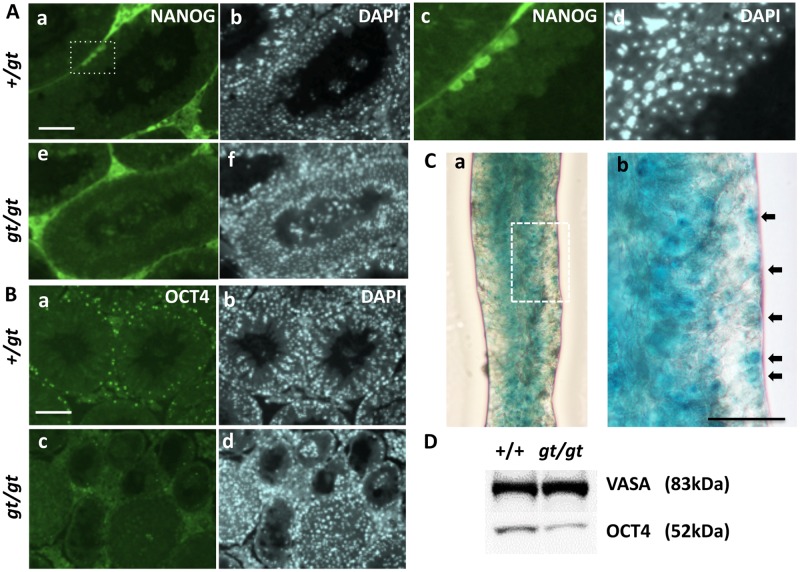
Apparent loss of NANOG/OCT4-expressing cells (putative SSCs) in the *Jmjd1C* ^*gt/gt*^ adult testis. A) Sections of littermate +/gt (a-d) and gt/gt (e-f) adult testes (4-month-old, stage XII) were stained with anti-NANOG. DAPI staining images corresponding to each immunostaining are shown in (b, d, f). Magnification of the broken line-edged frame indicated in (a) was shown in (c, d). B) Sections of littermate +/gt (a, b) and gt/gt (c, d) adult testes were stained with anti-OCT4. C) X-gal staining of the +/gt seminiferous tubules (a). Magnification of the broken line-edged frame indicated in (a) was shown in (b). Arrows indicate positive-stained cells attached to the basement membrane. Scale bars, 50 μm. D) Immunoblots of the +/+ and gt/gt testis extracts were double-stained with anti-VASA and anti-OCT4, and the resulting VASA (83 kDa) and OCT4 (52 kDa) bands were shown.

In this connection, an interesting finding was the existence of a rare subpopulation of undifferentiated spermatogonia expressing both NANOG and OCT4 that possibly corresponded to radiation-resistant SSCs in a certain quiescent state [26]. By immunohistochemical staining with anti-OCT4 and anti-NANOG, we detected OCT4-positive cells in spermatogonia residing in the outermost layer of the seminiferous tubule and extremely low numbers of NANOG-positive cells attached to the basement membrane of the *Jmjd1C*^*+/gt*^ testis (Fig 13A and 13B). Moreover, *Jmjd1C*-positive cells recognized by X-gal staining of the *Jmjd1C +/gt* seminiferous tubules were detected not only in the meiotic and postmeiotic cell layers but also in a specific portion of the spermatogonial cell layers as scattered cells on the basement membrane (Fig 13C). Additionally, extremely low numbers of strongly *Oct4-GFP*-positive cells were detected in a similar location in the *Oct4-GFP/Vasa-RFP* transgenic mouse testis (S6 Fig). In contrast, OCT4-positive cells were dramatically decreased, and NANOG-positive cells were barely detectable in the *Jmjd1C* ^*gt/gt*^ testis (Fig 13A and 13B). The reduction of OCT4 expression in the *Jmjd1C* ^*gt/gt*^ testis (less than half of the expression of the littermate controls) was validated by the immunoblotting analysis (Fig 13D).

**Fig 14:**
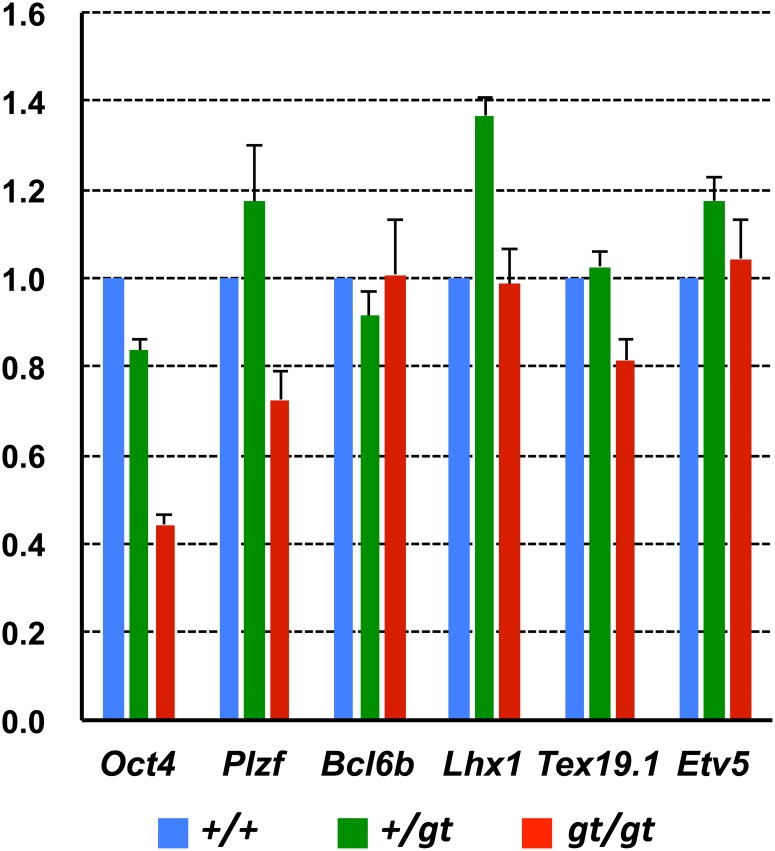
QRT-PCR analyses of SSC related genes between the +/+, +/gt, and gt/gt testes. Single-stranded cDNA was prepared from the +/+, +/gt and gt/gt testes of adult (2-month-old) littermate mice. Expression levels of selected genes related to spermatogonial stem cell (SSC) characteristics were compared. The values with SEM (n = 4) were the relative gene expression values in the +/+ testis (set as 1.0) after standardization with the β-actin expression level in each testis.

Several candidate genes important for the maintenance of SSC potential, such as *Bcl6b*, *Lhx1*, *Etv5* and *Tex19*.*1*, have been nominated based on findings that mouse SSCs can be cultured in a chemically defined medium containing GDNF (which is required for SSC self-renewal) and that withdrawal of GDNF from the medium and long-term culture in vitro leads to a loss of SSC potential [27, 28]. To evaluate the effect of *Jmjd1C* deficiency on SSC potential, the expression of selected genes related to SSC self-renewal and proliferation was compared between the *Jmjd1C* ^*gt/gt*^ testis and the littermate controls. As shown in Fig 14, most SSC potential genes showed no significant differences in their expression levels. The most remarkable reduction in the *Jmjd1C* ^*gt/gt*^ testis was detected in *Oct4* expression. Altogether with the finding that both in vivo and in vitro SSCs express *Oct4*, it is conceivable that *Oct4* expression under the control of JMJD1C is closely related to the maintenance of SSC self-renewal and/or survival similar to the pluripotency of ES cells.

## Discussion

### Multi-faceted functions of *Jmjd1C* during spermatogenesis

*Jmjd1C* is a member of the lysine demethylase (Kdm) 3 family, which consists of three members (*Jmjd1A*, *B*, and *C*). Among them, *Jmjd1A* is the first gene shown to play an essential role in the epigenetic regulation of spermatogenic gene expression [6]. The following studies have also revealed its multiple roles in metabolic homeostasis and sex determination through H3K9 demethylase activity and/or non-enzymatic activity [6–8, 29], raising the possibility that the KDM3 family has a common function not only as an epigenetic regulator of transcription but also as a fine modulator of the post-translational modification of non-histone proteins. Indeed, *Jmjd1C* showed no demethylation activity towards dimethylated H3K9 when dimethyl-peptides were used as the substrates [11], suggesting that the spermatogenic abnormalities found in the *Jmjd1C*^*gt/gt*^ testis were probably due to JMJD1C activities other than the demethylation of histone H3K9. Nevertheless, several findings showed a relationship between JMJD1C and H3K9 demethylation (i.e., the *P450e17* gene promoter in Leydig cells [30]). Similarly, the *Oct4* and *miR302* promoter regions in ES cells have been validated to be under the regulation of JMJD1C-dependent hypomethylation of H3K9 modification to retain their active states of transcription [31, 32]. The pluripotency-associated microRNA *miR302* represses the neural differentiation of ES cells, whose transcription is enhanced by the OCT4 and JMJD1C compound [31]. Moreover, we found that JMJD1C was associated with a restricted portion of chromatin in pachytene spermatocytes (Fig 2). Altogether, it seems likely that a cell-type specific cofactor(s) may be required to fulfill the JMJD1C demethylase activity towards methylated H3K9 in a locus-specific manner or that locus-specific association of enzymatically inactive JMJD1C is required to maintain the hypomethylated H3K9 state upon chromatin remodeling by blocking repressive methyl modification of the target loci. Here, a precedent study suggested that Jmj-C family proteins had a non-enzymatic function that was required for normal development (i.e., the H3K27 demethylases JMJD3 and UTX played a crucial role in general chromatin remodeling in differentiated cells that was entirely independent of their demethylase potential) [33]. Therefore, we can generalize that Jmj-C family proteins also have a non-catalytic function through interactions between lineage-specific transcription factors and remodeling complex components to keep the promoter region active in the epigenetic microenvironment.

JMJD1C has been shown to have the ability to demethylate non-histone proteins. For instance, as a core component of the DNA damage response (DDR) complex, JMJD1C binds to and demethylates MDC1 at K45, thereby promoting the MDC1-RNF8/RNF168 interaction required for the recruitment of RAP80-BRAC1 to DNA double-strand break sites [14]. The function of the DDR complex in spermatogenesis was visualized as the co-localization of JMJD1C and MDC1 at the XY body found in the spermatocyte nuclei (Fig 8). Additionally, MDC1 is critical for XY body formation and meiotic progression [15]. However, *Rnf8* and *Rnf168*, which encode E3 ubiquitin ligases, and *Jmjd1C* are dispensable for these processes [16, 19]. *Rnf8* functions upstream of *Rnf168*. *Rnf168*-deficient mice exhibit a defect in global nucleosome removal in the postmeiotic stages, whereas RNF8-dependent histone ubiquitination promotes H4K16 acetylation, which is an indirect trigger for the replacement of histones by protamines [16]. A reduced H4K16 acetylation level was also found in the *Jmjd1C*^*gt/gt*^ testis (Figs 10 and 11), leading to the speculation that *Jmjd1C* defects were at least partially due to the insufficient interaction between DDR components and that the mutual interactions within the DDR machinery played an important role in general chromatin remodeling through modulating H4K16 acetylation.

Regarding other possible candidate non-histone protein targets of the JMJD1C demethylase, we found several proteins that appeared to be hypermethylated in the *Jmjd1C*^*gt/gt*^ testis compared with the testes of control littermates. Among them, a 90 kDa protein appeared to be identical to a hypermethylated 90 kDa protein that co-immunoprecipitated with MDC1 from the *Jmjd1C*^*gt/gt*^ testis lysate (Fig 9A). Our findings that the cytoplasmic protein chaperone HSP90α, which is a highly methylated protein, was immunoprecipitated with anti-MDC1 (Fig 9B) and that both proteins were co-expressed in germ cells at the same spermatogenic stages (S4A Fig) strongly indicated that HSP90α was a possible target of the JMJD1C demethylase function. Moreover, previous studies showed that both HSP90α and JMJD1C co-immunoprecipitated with the histone methyltransferase WHISTLE, which raised a possibility that HSP90α had broad interacting activity with JmjC-family proteins, such as JMJD1A and KDM6A [29, 30]. Together, these results suggest that a protein-protein interaction between HSP90α and JMJD1C (belonging to the DDR complex) plays a crucial role in the modulation of mutual functions, probably by altering the methylation status.

Intriguingly, *Hsp90α*-deficient mice also exhibit male infertility [34]. HSP90α is methylated by SMYD2 and is involved in the organization of cytoskeletal components, such as γ-tubulin and β-actin, leading to the disorganization and abnormal shaping of spermatids observed in the *Hsp90α*-deficient testis [34, 35]. Moreover, a recent report revealed that the H3K9 demethylase activity of JMJD1A was dependent on the interaction with either HSP90α or HSP90β and that the *Jmjd1A* mutant showed cytoplasmic defects in cytoskeletal rearrangement during spermiogenesis in addition to the *Jmjd1A* defects that were reported previously [29]. Interestingly, because demethylation of the HSP90α K616 residue is not altered by the lack of *Jmjd1A*, another JmjC-containing protein may be responsible for the HSP90 demethylation. These results also support the possibility that HSP90α is a target of JMJD1C’s demethylase activity and raise a question as to whether the JMJD1C-dependent defect in the HSP90 machinery leads to the disorganized rearrangement and malformation of spermatids found in the *Jmjd1C*^*gt/gt*^ young testes. Altogether, it is quite comprehensible that JMJD1C serves as a scaffold to recruit various partners and promote mutual interactions by modulating their methylation statuses at each spermatogenic stages.

### Progressive loss of spermatogonial stem cells (SSCs)

*Jmjd1C* gene-trap mice in this study were backcrossed on to a 129/svj or Jcl/ICR background, whereas the complete null knockout mice in the previous study were analyzed on a C57BL/6 background [9]. It has been reported that several lines of *Jmjd1A* knockout mice show a drastic change of phenotype on the different genetic background [6–8, 36]. In contrast, although we can not deny a possibility that the difference between gene-trap and null-type knockout, and that of the genetic background causes a subtle phenotypic difference, the testis-restricted deficiency that asynchronous degradation of germ cell layers in the seminiferous tubules was common in both *Jmjd1C*-deficient mouse lines (Fig 4). In the null knockout mice, the progressive loss of undifferentiated spermatogonia was detected based on PLZF expression [9]. *Plzf* encodes a repressive transcription factor that is a well-known marker gene expressed in both SSCs and proliferating spermatogonia in the adult testis. Lack of *Plzf* function causes a progressive loss of spermatogonia without any differentiation defects, although the timing of degeneration appeared to be much earlier than the timing observed for the *Jmjd1C* defect [10]. Continuous proliferation of SSCs in vitro [referred to as germline stem cells (GSCs)] is maintained in a defined culture medium containing GDNF, LIF, EGF and bFGF. Stem cell potential is evaluated as a frequency of regenerated spermatogenesis based on transplantation into recipient testicular tubules in vivo. GSCs tend to lose their stem cell potential after long-term culture or withdrawal of GDNF. From this perspective, several genes, such as *Lhx1*, *Etv5* and *Bcl6b*, have been identified to be important for stem cell potential [27]. However, we found that these genes showed no significant differences between the *Jmjd1C*^*gt/gt*^ testis and the littermate control testis; instead, the most remarkable difference in the *Jmjd1C* ^*gt/gt*^ testis was a reduction in *Oct4* expression (Fig 14). GSCs express a set of iPS cell-inducing genes (*Oct4*, *Sox2*, *Myc* and *Klf4*), although the levels are somewhat lower than the levels in ES cells [37]. However, these cells do not express *Nanog*, which is in contrast to the ES and iPS cells [28]. Importantly, a few GSCs spontaneously dedifferentiate into pluripotent stem cells (referred to as multipotent GSCs) during the initiation of GSC cultures from postnatal testes [37].

A recent report that a small subpopulation of NANOG/OCT4-positive cells was found in the outermost cell layer attached to the basement membrane of seminiferous tubules in a specific spermatogenic stage was very intriguing [26] because it suggested that the conversion of cellular characteristics leading to NANOG expression might occur in both *in vitro* and *in vivo* self-renewing SSCs. NANOG-positive SSCs in the adult testis were also detected in this study but were undetectable in the *Jmjd1C*^*gt/gt*^ testis, probably due to the remarkable decrease of OCT4-positive cells (Fig 13). JMJD1C has been reported to be essential for the maintenance of pluripotency via promoting the active expression of *Oct4* and *Nanog* during ES cell proliferation. Additionally, up-regulation of *Jmjd1C* is required for the generation of completely reprogrammed iPS cells that express endogenous *Oct4* [32]. Provided that the self-renewal of SSCs is based on maintaining their potentially pluripotent state, it is reasonable to speculate that JMJ1C plays a critical role in maintaining the self-renewal and stem cell potential of SSCs via essentially the same function described for pluripotent cells.

Currently, JMJD1C is thought to play an extensive role related to various stem cell potentials. As described above, OCT4 is regarded as a functional partner of JMJD1C in pluripotent stem cells and probably also SSCs. Similarly, JMJD1C is assumed to participate in the maintenance of proliferative stem cells through association with lineage-specific transcription factors. Indeed, several reports have noted that JMJD1C is not required for the survival and self-renewal of leukemia stem cells. For instance, JMJD1C functions as a co-activator for Runx1-Runx1T1 in acute myeloid leukemia cell lines [38] and for HoxA9 in mixed lineage leukemia-AF9 and HoxA9-driven leukemia [39]. Therefore, it is conceivable that JMJD1C is recruited by partner transcription factors to their target genes and activates their expression by maintaining low H3K9 methylation levels, although whether JMJD1C acquires demethylase activity through association with a partner is not clear.

As shown in Fig 2, *Jmjd1C* expression was not restricted to the testis. Instead, testicular expression was at a relatively moderate level among adult tissues, and high level expression was primarily observed in the developing nervous system in embryos and neurons in the adult brain. Nevertheless, an apparent morphological or behavioral disorder was not found in the *Jmjd1C*^*gt/gt*^ adult mice, which might be due to a functional redundancy with other Jmj family genes. However, recent progress in the genetic analysis of human diseases noted possible causal relationships between *Jmjd1C* mutations and mental disorders as well as relationships with various cancers [25, 40]. For instance, *Jmjd1C* is a candidate gene for autism [41] because the breakpoint of chromosomal abnormality in the patient is located in the first intron of *Jmjd1C*. Moreover, *Jmjd1C* mutations have been identified in a subpopulation of Rett syndrome patients [42], suggesting that *Jmjd1C* could be related to higher order brain functions, such as mental faculties.

Further detailed investigations to clarify the partners and targets of JMJD1C functions in each cell lineage will provide important clues to understanding the action mechanism of the JMJD1C-dependent regulatory network and to develop novel therapeutic targets for cancers and mental disorders.

## Supporting Information

S1 Fig Ratio of two variants during testis development and detection of JMJD1C in haploid cells. A) Ratio (%) of the short and long variant expression levels in the testes on the indicated days after birth are presented. The values were based on the data shown in Fig 5. B) Immunoblotting detection of the JMJD1C protein in 1N, 2N and 4N cells, which were fractionated from the adult testis (6-month-old) by flow-cytometry using Hoechst dye vital staining. Approximately 15 μg of the protein extract of each fraction was analyzed. JMJD1C was detected in both the 1N (spermatid) and 4N (primarily spermatocyte) cell fractions but was barely detected in the 2N (spermatogonia and somatic cells) fraction. The detection of α-TUBULIN and spermatocyte-specific SCP3 with antibodies was used as the standard control.
(TIF)

S2 Fig Detection of *Jmjd1C* expression in the *Jmjd1C*-deficient testis. A) Using single-stranded cDNA prepared from the +/+, +/gt and gt/gt adult testes, RT-PCR was performed with primer pairs located at the indicated exons numbers as shown in the upper panel. B) Immunoblotting detection of JMJD1C in protein extracts from the +/+, +/gt and gt/gt adult testes (2-month-old). Protein bands detected in the same membrane using antibodies against VASA, HSC70t and histone H3 were presented as the positive controls.
(TIF)

S3 Fig Ultrastructural abnormality found in the spermatids of the *Jmjd1C*-deficient testis. Electron micrographs of high-magnification views of condensed spermatids from the *Jmjd1C* heterozygous (+/gt) (a) and homozygous (gt/gt) testes (b) of 3-month-old littermate mice are presented. Spermatids of the homozygote appear to be disorganized in orientation and have fewer condensed nuclei compared with the heterozygote. Scale bar in (a), 2 μm
(TIF)

S4 Fig Immunohistochemical detection of selected proteins in the *Jmjd1C*-deficient testis. A) Sections of littermate +/+ (a-c) and gt/gt (d-f) adult (4-month-old) testes (stage X) were double-stained with anti-SCP3 (a, d) and anti-MDC1 (b, e). (c) and (f) are DAPI staining of the same fields as (a) and (d), respectively. Bar in (a) for (a-f), 20 μm. B) Sections of littermate +/+ (a-c) and gt/gt (d-f) adult (4-month-old) testes were double-stained with anti-SCP3 (a, d) and anti-RNF8 (b, e). (c) and (f) are DAPI staining of the same fields as (a) and (d), respectively. (g) and (h) are merged images of magnification views of the broken line-edged frame in (a+b) and (d+e), respectively. Scale bars in (a) for (a-f), 50 μm and in (g) for (g, h), 20 μm. C) Sections of littermate +/gt (a-c) and gt/gt (d-f) adult (4-month-old) testes were double-stained with anti-HSC70t (a, d) and anti-methylated lysine (Kme) (b, e). DAPI staining images corresponding to each immunostainings are shown in (c, f). Bar in (a) for (a-f), 100 μm. D) Sections of littermate +/+ (a-c) and gt/gt (d-f) adult (4-month-old) testes were double-stained with anti-SCP3 (a, d) and anti-MOF (b, e). (c) and (f) are DAPI staining of (a) and (d), respectively. Bar in (a) for (a-f), 50 μm.
(TIF)

S5 Fig Detection of specific reactants by immunoblotting using selected antibodies. Approximately 15 μg protein extracts from +/+ testes (4-month-old) were applied on SDS-PAGE (10–20%). The western-blotted membranes were separately reacted with antibodies against HSC70t, VASA, histone H3 (H3) and acetylated H4K16 (H4K16Ac) and then detected with HRP-linked anti-rabbit IgG and an ECL detection reagent.
(TIF)

S6 Fig OCT4-expressing cells localized beneath the basal membrane of the seminiferous tubule. Testicular tubules were prepared from an adult (8-month-old) transgenic mouse carrying *Oct4-GFP* and *Vasa-RFP* [43]. Strong GFP-positive cells with *Oct4* expression were observed just inside the basement membrane only in a restricted area of the seminiferous tubules. Merged GFP and phase contrast (a) or RFP and phase contrast (b) images in the same field are presented. Scale bar, 50 μm.
(TIF)

S1 Table Primers used in this study. (DOCX)

S2 Table Antibodies used in this study. (DOCX)
