# Supplementary figures and images for: JMJD1C Exhibits Multiple Functions in Epigenetic Regulation during Spermatogenesis
Source: PLoS One. 2016 Sep 20;11(9):e0163466. doi: 10.1371/journal.pone.0163466 (PMC5029890; doi:10.1371/journal.pone.0163466)

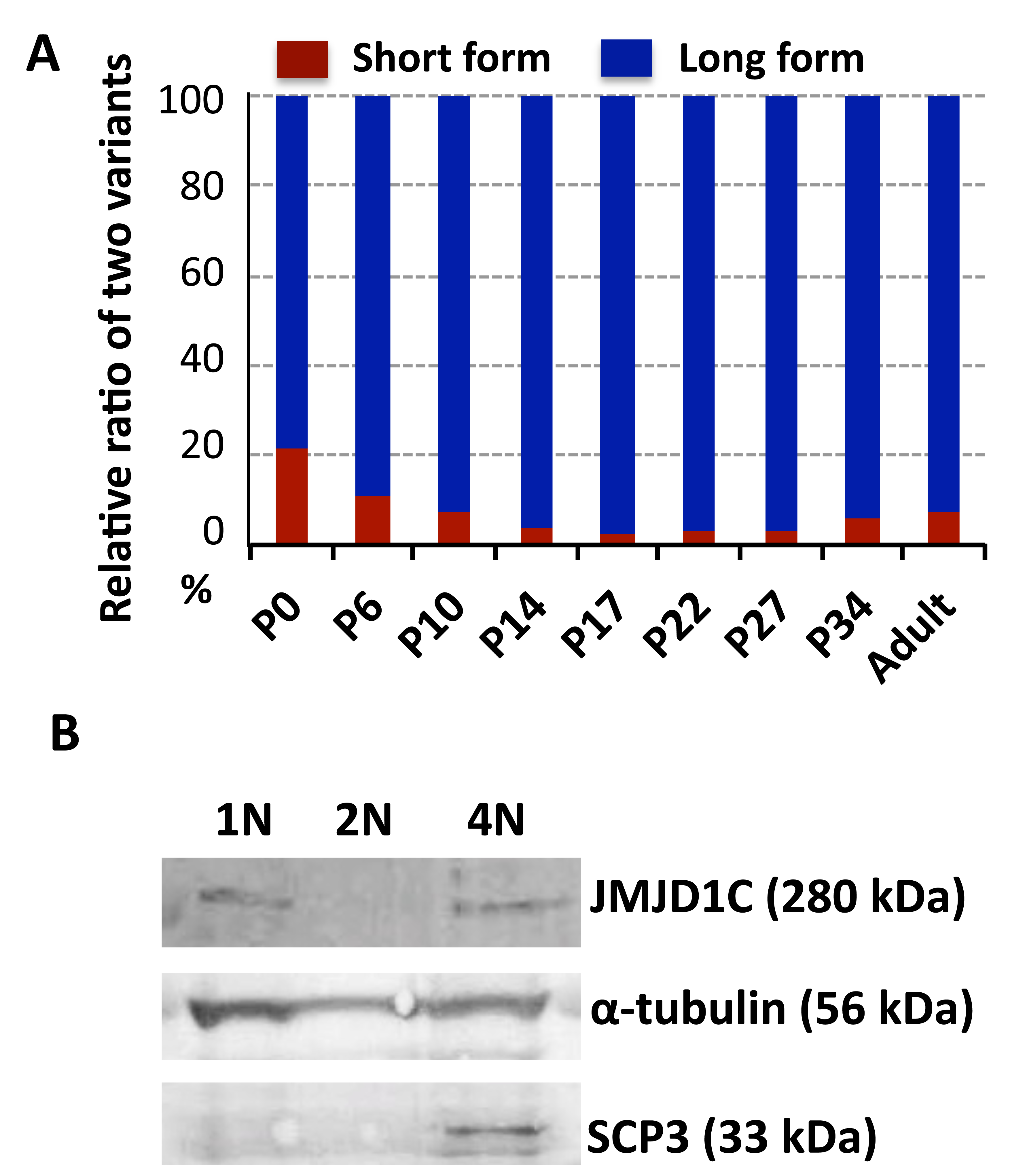

Supplement: S1 Fig — A) Ratio (%) of the short and long variant expression levels in the testes on the indicated days after birth are presented. The values were based on the data shown in Fig 5. B) Immunoblotting detection of the JMJD1C protein in 1N, 2N and 4N cells, which were fractionated from the adult testis (6-month-old) by flow-cytometry using Hoechst dye vital staining. Approximately 15 μg of the protein extract of each fraction was analyzed. JMJD1C was detected in both the 1N (spermatid) and 4N (primarily spermatocyte) cell fractions but was barely detected in the 2N (spermatogonia and somatic cells) fraction. The detection of α-TUBULIN and spermatocyte-specific SCP3 with antibodies was used as the standard control. (TIF) [file pone.0163466.s001.tif]

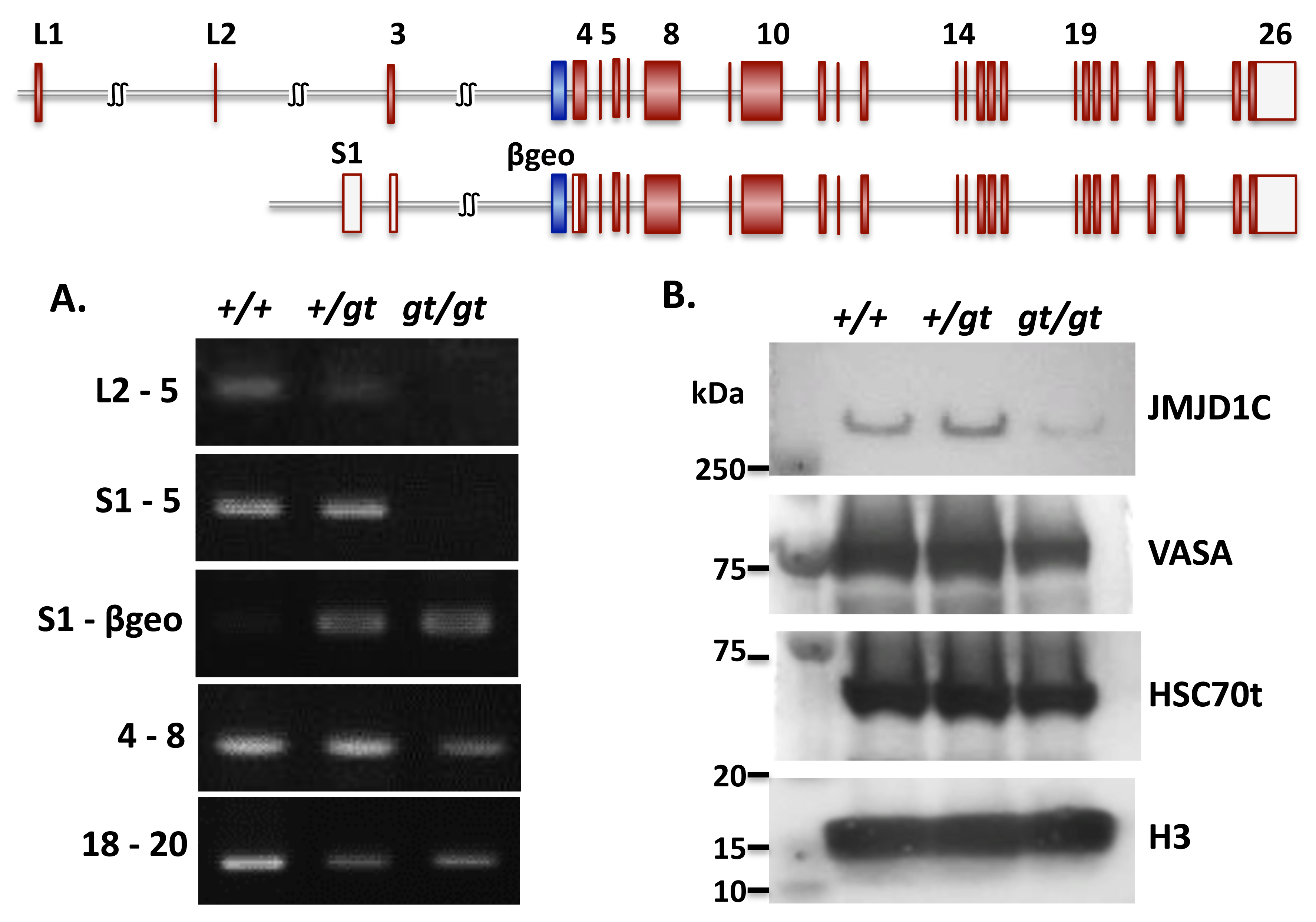

Supplement: S2 Fig — A) Using single-stranded cDNA prepared from the +/+, +/gt and gt/gt adult testes, RT-PCR was performed with primer pairs located at the indicated exons numbers as shown in the upper panel. B) Immunoblotting detection of JMJD1C in protein extracts from the +/+, +/gt and gt/gt adult testes (2-month-old). Protein bands detected in the same membrane using antibodies against VASA, HSC70t and histone H3 were presented as the positive controls. (TIF) [file pone.0163466.s002.tif]

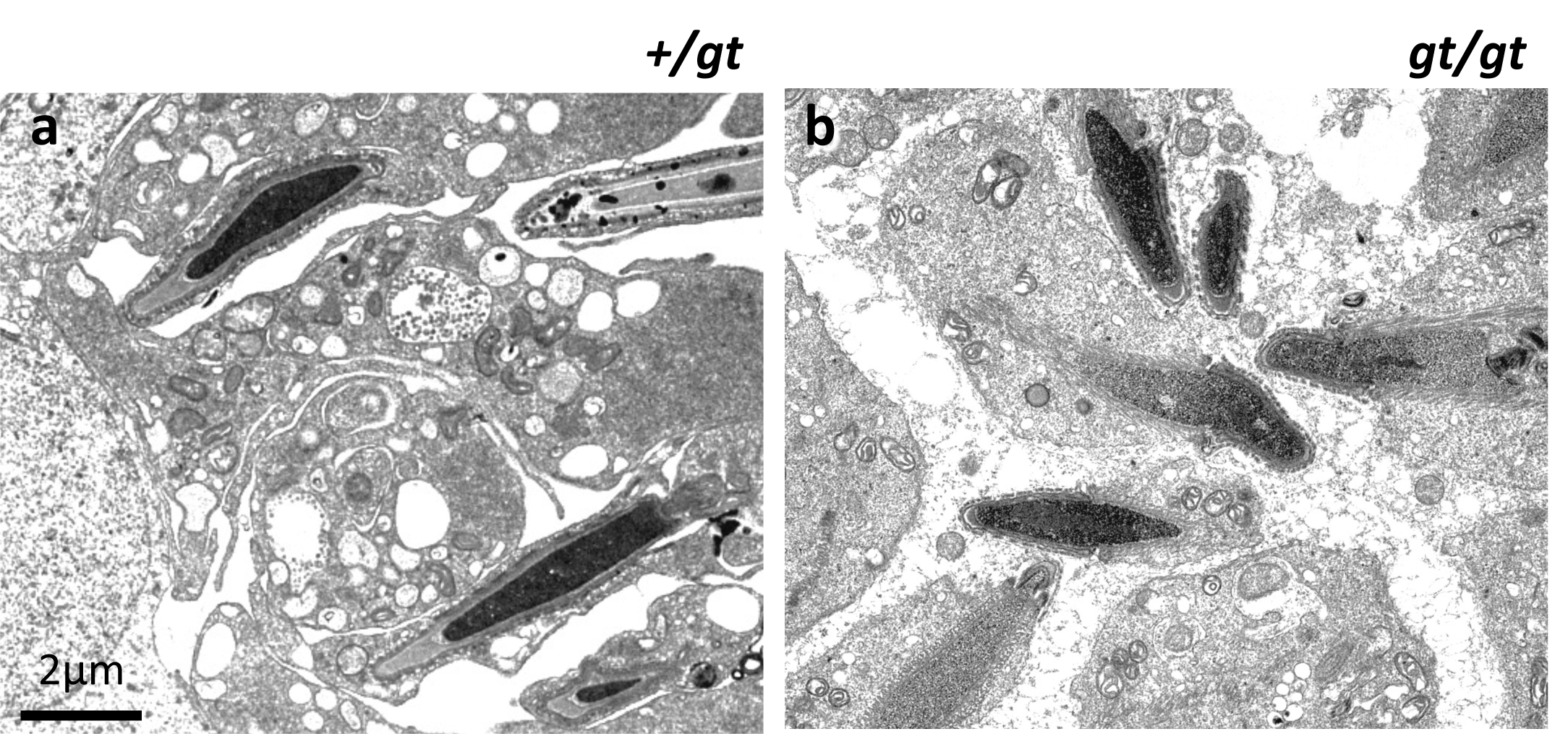

Supplement: S3 Fig — Electron micrographs of high-magnification views of condensed spermatids from the Jmjd1C heterozygous (+/gt) (a) and homozygous (gt/gt) testes (b) of 3-month-old littermate mice are presented. Spermatids of the homozygote appear to be disorganized in orientation and have fewer condensed nuclei compared with the heterozygote. Scale bar in (a), 2 μm (TIF) [file pone.0163466.s003.tif]

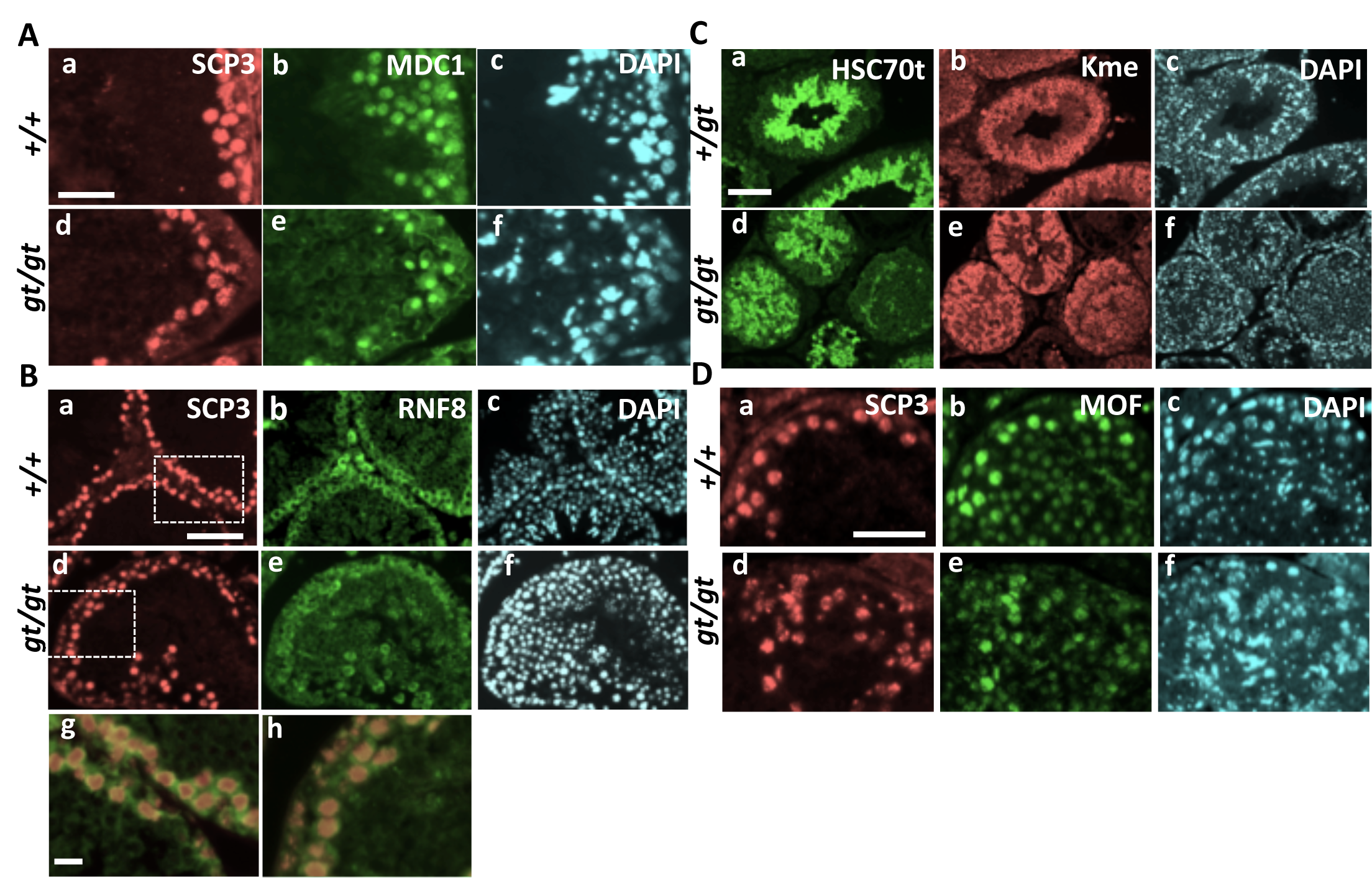

Supplement: S4 Fig — A) Sections of littermate +/+ (a-c) and gt/gt (d-f) adult (4-month-old) testes (stage X) were double-stained with anti-SCP3 (a, d) and anti-MDC1 (b, e). (c) and (f) are DAPI staining of the same fields as (a) and (d), respectively. Bar in (a) for (a-f), 20 μm. B) Sections of littermate +/+ (a-c) and gt/gt (d-f) adult (4-month-old) testes were double-stained with anti-SCP3 (a, d) and anti-RNF8 (b, e). (c) and (f) are DAPI staining of the same fields as (a) and (d), respectively. (g) and (h) are merged images of magnification views of the broken line-edged frame in (a+b) and (d+e), respectively. Scale bars in (a) for (a-f), 50 μm and in (g) for (g, h), 20 μm. C) Sections of littermate +/gt (a-c) and gt/gt (d-f) adult (4-month-old) testes were double-stained with anti-HSC70t (a, d) and anti-methylated lysine (Kme) (b, e). DAPI staining images corresponding to each immunostainings are shown in (c, f). Bar in (a) for (a-f), 100 μm. D) Sections of littermate +/+ (a-c) and gt/gt (d-f) adult (4-month-old) testes were double-stained with anti-SCP3 (a, d) and anti-MOF (b, e). (c) and (f) are DAPI staining of (a) and (d), respectively. Bar in (a) for (a-f), 50 μm. (TIF) [file pone.0163466.s004.tif]

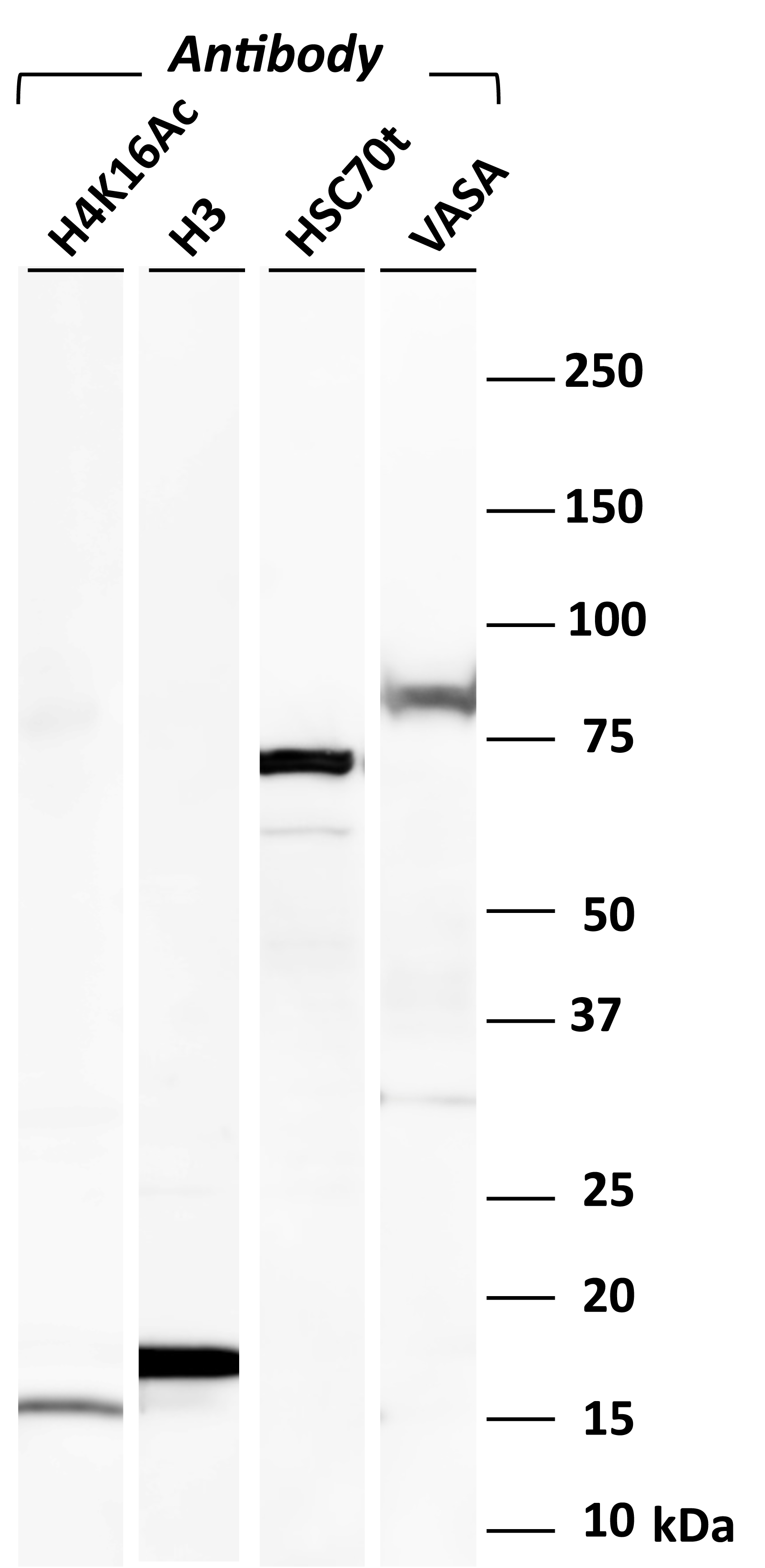

Supplement: S5 Fig — Approximately 15 μg protein extracts from +/+ testes (4-month-old) were applied on SDS-PAGE (10–20%). The western-blotted membranes were separately reacted with antibodies against HSC70t, VASA, histone H3 (H3) and acetylated H4K16 (H4K16Ac) and then detected with HRP-linked anti-rabbit IgG and an ECL detection reagent. (TIF) [file pone.0163466.s005.tif]

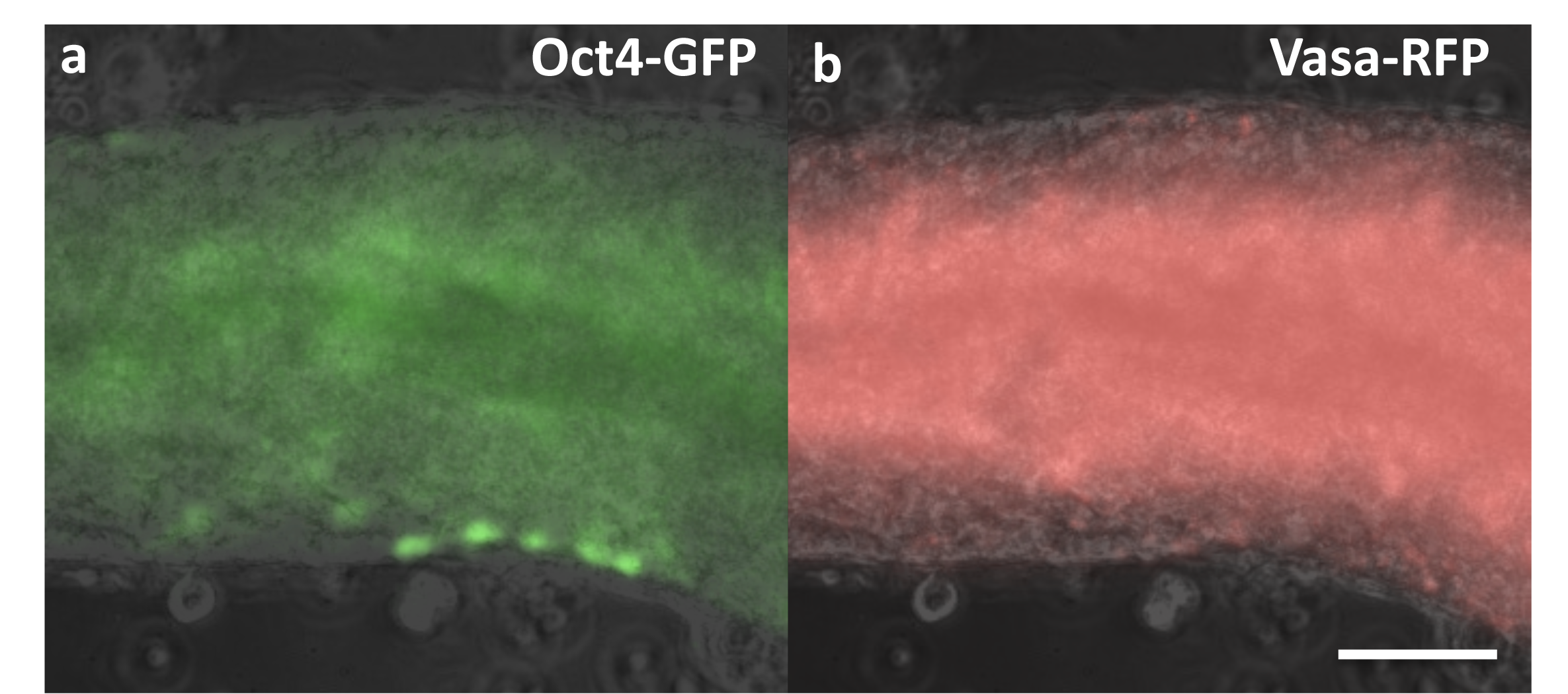

Supplement: S6 Fig — Testicular tubules were prepared from an adult (8-month-old) transgenic mouse carrying Oct4-GFP and Vasa-RFP [43]. Strong GFP-positive cells with Oct4 expression were observed just inside the basement membrane only in a restricted area of the seminiferous tubules. Merged GFP and phase contrast (a) or RFP and phase contrast (b) images in the same field are presented. Scale bar, 50 μm. (TIF) [file pone.0163466.s006.tif]
