# Supplementary material for: JMJD1C Exhibits Multiple Functions in Epigenetic Regulation during Spermatogenesis
Source: PLoS One. 2016 Sep 20;11(9):e0163466. doi: 10.1371/journal.pone.0163466 (PMC5029890; doi:10.1371/journal.pone.0163466)
Supplement: S1 Table — (DOCX) [file pone.0163466.s007.docx]

**S1 Table. Primers used in this study**

| Name | Sequence (5'→3') | Use |
| --- | --- | --- |
| qβ-Actin forward | TATGGAATCCTGTGGCATCC | QRT-PCR |
| qβ-Actin reverse | ACATCTGCTGGAAGGTGGAC | QRT-PCR |
| qTnp1 forward | GAGAGGTGGAAGCAAGAGAAAA | QRT-PCR |
| qTnp1 reverse | CCCACTCTGATAGGATCTTTGG | QRT-PCR |
| qTnp2 forward | GAAGGGAAAGTGAGCAAGAGAA | QRT-PCR |
| qTnp2 reverse | GCATAGAAATTGCTGCAGTGAC | QRT-PCR |
| qVasa forward | GTATTCATGGTGATCGGGAGCAG | QRT-PCR |
| qVasa reverse | CAACAAGAACTGGGCACTTTCCA | QRT-PCR |
| qPrm1 forward | ACACAGGCGCTGCTTCGTAA | QRT-PCR |
| qPrm1 reverse | GTGATGGTGCCTCCACATTTCCT | QRT-PCR |
| qAcrosin forward | TGTCCGTGGTTGCCAGGATAACA | QRT-PCR |
| qAcrosin reverse | AATCCGGGTACCTGTTGTGAGTT | QRT-PCR |
| qPlzf forward | TGCGCAGCTATATTTGCAGT | QRT-PCR |
| qPlzf reverse | ATAGGGCTTCTCCCCTGTGT | QRT-PCR |
| qSycp3 forward | ATGAATGTGTTGCAGCAGTG | QRT-PCR |
| qSycp3 reverse | GCTCGTGTATCTGTTTGATTGC | QRT-PCR |
| qDmc1 forward | TTTCAAGACATTGACCTGTTGC | QRT-PCR |
| qDmc1 reverse | TAAGCTTGTTGGCTGCCTCT | QRT-PCR |
| qRnf8_forward | ACTTGCTGGAGAACGAGCTC | QRT-PCR |
| qRnf8 reverse | CCAGGGAGTTGGTTCTGGAC | QRT-PCR |
| qRnf168 forward | CCATGCTTCCAGTCCACTGT | QRT-PCR |
| qRnf168 reverse | CCTCAACTCCCCAGGTTCAC | QRT-PCR |
| qMof forward | CCCAGAGAAGCCACTGAGTG | QRT-PCR |
| qMof reverse | GGCCCTTCCAGTACTTGACC | QRT-PCR |
| qChd5 forward | TGCAACCATCCGTACCTCTTCC | QRT-PCR |
| qChd5 reverse | TCAGCACTCTGTGCCCTTCATC | QRT-PCR |
| qBaz1a forward | GTCGTTCACGCTTGCATGAA | QRT-PCR |
| qBaz1a reverse | ATCAGCACTGCTCAGGTGTC | QRT-PCR |
| qBrdt forward | CATGCATGCCACCTTACAACA | QRT-PCR |
| qBrdt reverse | AGGCTTTTTCCTTGGGTTTTC | QRT-PCR |
| qPA200 forward | ATTCAGCCTGTCCTCTTGGC | QRT-PCR |
| qPA200 reverse | CCCAGGCAATGCTCTCATCA | QRT-PCR |
| qNat8f4 forward | CAGCACATCTTTCCACAGTG | QRT-PCR |
| qNat8f4 reverse | CTTCTCCTGGTACTGTCGGATG | QRT-PCR |
| qOct4 forward | CCAATCAGCTTGGGCTAGAG | QRT-PCR |
| qOct4 reverse | CTGGGAAAGGTGTCCCTGTA | QRT-PCR |
| qMdc1 forward | GGGCAGCTACGTCTCTTCAG | QRT-PCR |
| qMdc1 reverse | GCTCACTCCAGGAGGTAGGA | QRT-PCR |
| qBcl6b forward | CAATCTGAATGAGCTGCGCC | QRT-PCR |
| qBcl6b reverse | GCCTGGACTACGTGTTCCAT | QRT-PCR |
| qLhx1 forward | CAACCTGACCGAGAAGTGCT | QRT-PCR |
| qLhx1 reverse | CGGTGGAGAGCTGCTTGTTA | QRT-PCR |
| qTex19.1 forward | TCTGGAAGCTCAAGCCTGTC | QRT-PCR |
| qTex19.1 reverse | AAAGCTGCCTTGAAGCAAGC | QRT-PCR |
| qEtv5_forward | AAGATCAAACGGGAGCTGCA | QRT-PCR |
| qEtv5 reverse | GCGGGGTTAATGGCTTGAAC | QRT-PCR |
| qJmjd1C forward | CTAGAACCACAGAATGTCGA | QRT-PCR |
| qJmjd1C reverse | TGAGCACGTGTATAATGACC | QRT-PCR |
| Jmjd1C Long Exon2 forward | CCAAAAGGAAGGACCCTAGC | QRT-PCR, RT-PCR |
| Jmjd1C Short Exon1 forward | CAATGACGGCGCTGCAAGTG | QRT-PCR, RT-PCR |
| Jmjd1C Exon3 reverse | TGGTATGGCTGAAAGGCACT | QRT-PCR, RT-PCR |
| Jmjd1C Exon4 forward | CCAGGAGATTTTTATGCAAG | RT-PCR |
| Jmjd1C Exon5 reverse | ACTGGGTGGCAGAATCTTGT | RT-PCR |
| Jmjd1C Exon8 reverse | CACCACGTGCTTGTTCTTAC | RT-PCR |
| Jmjd1C Exon18 forward | ACCTCAAGTGGCAAAGGTGG | RT-PCR |
| Jmjd1C Exon20 reverse | CATATCTTGTTGGCATCATG | RT-PCR |
| β-geo reverse | TGGTTTTCGGGACCTGGGAC | RT-PCR |
| Jmjd1C GT_forward | ATACGGGATAATACCGCGCCAC | Genotyping |
| Jmjd1C Common_reverse | AAGTCACTCAGGTACCGAAGG | Genotyping |
| Jmjd1C WT_forward | GCTGTTATAATACCCTGGTG | Genotyping |
