# Supplementary material for: JMJD1C Exhibits Multiple Functions in Epigenetic Regulation during Spermatogenesis
Source: PLoS One. 2016 Sep 20;11(9):e0163466. doi: 10.1371/journal.pone.0163466 (PMC5029890; doi:10.1371/journal.pone.0163466)
Supplement: S2 Table — (DOCX) [file pone.0163466.s008.docx]

**S2 Table. Antibodies used in this study**

IHC: immunohistochemistry, WB: western-blot, IP: immunoprecipitation

Rb:rabbit, Ms:mouse, Gt: goat, Dn; donkey, Sh; sheep

| Antibody (species) | Manufacturer (code or reference) | Use (dilution rate) |
| --- | --- | --- |
| anti-VASA (MVH)(Rb) | Self-made, [12] | WB (10^-5^), IHC (10^-4^) |
| anti-HSC70t (Rb) | Self-made, [13] | WB (1/4000), IHC (1/1000) |
| anti-SCP3 (Ms) | Abcam (ab97672) | WB (1/1000), IHC (1/1000) |
| anti-JMJD1C (Rb) | Abcam (ab31215) | WB (1/1000), IHC (1/1000) |
| anti-MDC1 (Rb) | Abcam (ab41951) | WB (1/1000), IP, IHC (1/1000) |
| anti-RNF8 (Rb) | Abcam (ab105362) | WB (1/1000), IHC (1/500) |
| anti-MOF (Rb) | Abcam (ab200660) | WB (1/1000), IHC (1/1000) |
| anti-TNP1 (Rb) | Abcam (ab73135) | IHC (1/1000) |
| anti-OCT4 (Rb) | Abcam (ab19857) | WB (1/1000), IHC (1/500) |
| anti-HSP90α (Rb) | Abcam (ab79849) | WB (1/2000), IHC (1/1000) |
| anti-NANOG (Ms) | Cell Signaling Tech (4893) | IHC (1/500) |
| anti-Protamine1(Gt) | Santa Cruz Biotech (sc-23107) | IHC (1/500) |
| anti-Methylated Lysine (Rb) | Abcam (ab23366) | WB (1/1000), IHC (1/1000) |
| anti-Acetylated H4K16(Rb) | Abcam (ab109463) | WB (1/1000), IHC (1/1000) |
| anti-Histone H3 (Rb) | Abcam (ab1791) | WB (1/4000) |
| anti-βgalactosidase (Ms) | Promega (Z3781) | IHC (1/1000) |
| [Alexa Fluor-488] anti-rabbit IgG (Gt) | Thermo Fisher Scientific (A-11034) | IHC (1/1000) |
| [Alexa Fluor-568] anti-rabbit IgG (Gt) | Thermo Fisher Scientific (A-11036) | IHC (1/1000) |
| [Alexa Fluor-488] anti-mouse IgG (Gt) | Thermo Fisher Scientific (A-11029) | IHC (1/1000) |
| [Alexa Fluor-568] anti-mouse IgG (Gt) | Thermo Fisher Scientific (A-11031) | IHC (1/1000) |
| [Alexa Fluor-488] anti-goat IgG (Dn) | Thermo Fisher Scientific (A-11055) | IHC (1/1000) |
| [Alexa Fluor-568] anti-goat IgG (Dn) | Thermo Fisher Scientific (A-11057) | IHC (1/1000) |
| [HRP] anti-rabbit IgG (Dn) | GE-HealthCare (NA934VS) | WB (1/2000) |
| [HRP] anti-mouse IgG (Sh) | GE-HealthCare (NA931VS) | WB (1/2000) |
